# Generating Phenotypic Diversity in a Fungal Biocatalyst to Investigate Alcohol Stress Tolerance Encountered during Microbial Cellulosic Biofuel Production

**DOI:** 10.1371/journal.pone.0077501

**Published:** 2013-10-16

**Authors:** Rosanna C. Hennessy, Fiona Doohan, Ewen Mullins

**Affiliations:** 1 Department of Crop Science, Teagasc Research Centre, Oak Park, Carlow, Ireland; 2 Molecular Plant-Microbe Interactions Laboratory, School of Biology and Environmental Science, University College Dublin, Dublin, Ireland; University of Kentucky, United States of America

## Abstract

Consolidated bioprocessing (CBP) of lignocellulosic biomass offers an alternative route to renewable energy. The crop pathogen *Fusarium oxysporum* is a promising fungal biocatalyst because of its broad host range and innate ability to co-saccharify and ferment lignocellulose to bioethanol. A major challenge for cellulolytic CBP-enabling microbes is alcohol inhibition. This research tested the hypothesis that *Agrobacterium tumefaciens* - mediated transformation (ATMT) could be exploited as a tool to generate phenotypic diversity in *F. oxysporum* to investigate alcohol stress tolerance encountered during CBP. A random mutagenesis library of gene disruption transformants (*n*=1,563) was constructed and screened for alcohol tolerance in order to isolate alcohol sensitive or tolerant phenotypes. Following three rounds of screening, exposure of select transformants to 6% ethanol and 0.75% *n*-butanol resulted respectively in increased (≥11.74%) and decreased (≤43.01%) growth compared to the wild –type (WT). Principal component analysis (PCA) quantified the level of phenotypic diversity across the population of genetically transformed individuals and isolated candidate strains for analysis. Characterisation of one strain, Tr. 259, ascertained a reduced growth phenotype under alcohol stress relative to WT and indicated the disruption of a coding region homologous to a putative sugar transporter (FOXG_09625). Quantitative PCR (RT-PCR) showed FOXG_09625 was differentially expressed in Tr. 259 compared to WT during alcohol-induced stress (*P*<0.05). Phylogenetic analysis of putative sugar transporters suggests diverse functional roles in *F. oxysporum* and other filamentous fungi compared to yeast for which sugar transporters form part of a relatively conserved family. This study has confirmed the potential of ATMT coupled with a phenotypic screening program to select for genetic variation induced in response to alcohol stress. This research represents a first step in the investigation of alcohol tolerance in *F. oxysporum* and has resulted in the identification of several novel strains, which will be of benefit to future biofuel research.

## Introduction

Lignocellulosic biomass is an abundant feedstock and attractive source of sugars for biofuel production. Large-scale utilisation is however challenged by the general lack of low-cost technologies that can overcome cellulose recalcitrance [1]. One potential route to eco-friendly sustainable energy production is the consolidated bioprocessing (CBP) of biomass into biofuels [2].

 Several yeasts (e.g. *Saccharomyces cerevisiae*, *Pichia stipitis*, *Candida shehatae*, *Pachysolen tannophilus*) and bacteria (e.g. *Escherichia coli*, *Klebsiella oxytoca*, *Zymomomas mobilis*) have been engineered for CBP ethanol production however their capacity to secrete saccharification and fermentation enzymes at sufficient yield remains an obstacle [3-7]. In contrast, filamentous fungi including *Trichoderma* sp.*, Neurospora* sp.*, Aspergillus* sp.*, Monilinia* sp.*, Rhizopus sp., Mucor sp., Paecilomyces* sp. and *Fusarium* sp., possess a large repertoire of lignocellulolytic enzymes due to their co-evolution with plants, and can convert released plant-derived sugars into ethanol [7–10]. In particular, the broad host range phytopathogen *Fusarium oxysporum* [11] can degrade and produce ethanol from various cellulosic substrates (e.g. untreated and pre-treated straw [12,13], brewer’s spent grain [14], potato waste [15]). Previous work [13] identified *F. oxysporum* strain 11C as a promising microbial biocatalyst capable of producing high bioethanol yields from delignified wheat straw.

 At present no organism can ferment cellulosic materials to ethanol at rates and titres necessary to achieve economic feasibility [5]. CBP requires robust microbes able to: (i) degrade lignocellulosic materials in the absence of supplementary exogenous enzymes, (ii) utilise at high efficiency both hexose and pentose sugars including their conversion into high-titer ethanol and (iii) tolerate toxic compounds notably the primary alcohol product formed during fermentation [16]. Whereas the endogenous ability of *F. oxysporum* to tolerate inhibitory compounds encountered during CBP including lignocellulosic hydrosylates (carboxylic acids, phenolic compounds, furan derivatives) [17] and the fermentation by-product acetic acid [18] has been reported, a knowledge deficit exists in regards to *F. oxysporum*’s capability to tolerate ethanol.

 Ethanol stress affects cell growth and viability in addition to productivity [19,20]. While other compounds compatible as biofuels such as *n*-butanol offer advantages over ethanol (e.g. high energy content and miscibility with gasoline), production is limited by the sensitivity of native producers such as *Clostridium sp.* to the alcohol [21]. This has led to the investigation of alternative butanol production hosts [22-24], prompting the investigation of *n*-butanol (hereafter referred to as butanol) tolerance in addition to ethanol in this study. For the cost-effective and commercially viable production of biofuels, product yields must exceed native microbial tolerance levels [25] therefore engineering of stress tolerant strains is critical to achieve high survival and production rates [25,26].

 Several strategies have been investigated to improve alcohol tolerance in yeast and bacteria, including gene over-expression or random knockout libraries, genome shuffling and transcriptional or translational engineering [27]. For filamentous fungi ATMT has established itself as a valuable and powerful tool for genetic studies, greatly facilitating the identification and analysis of genes involved in complex biological processes such as host pathogenicity [28] and, as we hypothesise here, in regards to alcohol tolerance. As the gram-negative soil bacterium *A. tumefaciens* facilitates the stable transfer and integration of the tumour inducing (Ti) plasmid segment (known as T- DNA) into the targeted host cell [29], ATMT holds significant advantages over more traditional methods of gene disruption. For example, *A. tumefaciens*’ broad host range, its potential to transform a range of starting materials and its propensity for low copy number insertion events [30]. ATMT also provides an ability to ‘tag’ the disrupted gene(s) thereby facilitating their identification via genome walking. In the case of *F. oxysporum* this is further assisted by the recent availability of the organism’s genome sequence [31] (http://www.broadinstitute.org).

 In this study, we hypothesised that ATMT could be exploited as a tool to generate significant degrees of phenotypic diversity in *F. oxysporum* strain 11C in response to alcohol stress. To this end, a modified ATMT protocol was established to generate a library of random gene disruption transformants, which underwent a three tier physiological evaluation. As a result, the initial population of *F. oxysporum* transformants was reduced from 1,563 to 29 individuals, which were subjected to ethanol tolerance screening and cross-resistance to butanol. The level of recorded phenotypic variation confirmed the occurrence of ATMT-derived genotypes with several *F. oxysporum* transformants identified for future analysis. To the best of our knowledge this is the first report detailing the use of random insertional mutagenesis to investigate alcohol tolerance in a fungal CBP agent.

## Materials and Methods

### Origin and maintenance of fungi

*Fusarium oxysporum* strain 11C originated from Irish soils as previously described [13]. For ATMT, *F. oxysporum* was sub-cultured onto potato dextrose agar (PDA; Difco, UK) plates, incubated at 25°C for 5 days prior to producing fungal conidial inoculum in Mung bean broth [32].

### Plasmid and ATMT transformation

The binary vector used for fungal gene disruption was pSK1019, which is equipped with the *hph* antibiotic resistance marker gene under the control of an *Aspergillus nidulans TrpC* promoter and was donated by Professor Seogchan Kang (The Pennsylvania State University, USA). *Agrobacterium tumefaciens* strain AGL-1 was transformed with the binary vector pSK1019 [33] and ATMT of *F. oxysporum* strain 11C was optimised based on a previously published protocol [34] (see Methods S1). Three independent ATMTs of *F. oxysporum* strain 11C were conducted. 

### Molecular analysis of transformants

For PCR analysis, fungal genomic DNA was extracted from mycelia using a modified method [35] (see Methods S1). DNA was quantified using a Qubit^TM^ Quant-iT assay (Invitrogen, U.S) and stored at -80°C. PCR was used to detect the presence/absence of the *hph* transgene in DNA extracts from putative transformants (see Methods S1). For Southern blot analysis, genomic DNA was extracted [36] and quantified using a NanoDrop® ND-1000 Spectrophotometer (NanoDrop Technologies, USA). Southern blot analysis was used to determine plasmid copy number in transformants (see Methods S1). 

### Primary alcohol tolerance screen

A total of 1,563 putatively transformed hygromycin-resistant colonies were isolated into microplates (96-well) containing 200 μl minimal medium (supplemented with hygryomcinB, 60 μg ml^-1^, Sigma, UK) per well [37] which were then sealed and incubated at 25°C for 3 days and subsequently used as inoculum for primary screening of alcohol tolerance. For the primary screen (first of three), 96-well plates were prepared containing 140μl minimal medium [37] per well supplemented with six treatments either hygromycinB (60 μg ml^-1^), no ethanol or 0.5, 6.0 or 10% vv^-1^ ethyl alcohol (Sigma, UK) plus or minus hygromycinB (60 μg ml^-1^; Sigma, UK). To target different response levels, low (0.5% vv^-1^ ethanol), medium (6% vv^-1^ ethanol) and high (10% vv^-1^) ethanol concentrations were then selected. Wells were inoculated with 10 μl of fungal material (one well per treatment per transformant/wild type fungus; plate layout is shown in Figure S1A). Plates were sealed with a lid and parafilm and incubated at 25°C with fungal growth (OD_600nm_) measured after both 48 and 96 hours using a spectrophotometer (Safire^2^, Tecan, Austria). Putative transformants with a ≥ 2-fold higher OD_600nm_ in 10% (vv^-1^) ethanol relative to wild type fungus 11C were purified prior to secondary screening. This primary screening of the transformant collection was conducted once followed by the selection of putative transformants for purification and graduation to the secondary screening (see Methods S1).

### Secondary alcohol tolerance screen

Conidial suspensions of the *F. oxysporum* 11C wild type strain and transformants of interest (20 μl from the from monoconidial 20% (vv-^1^) -80°C glycerol stock) were inoculated into 220 μl minimal medium in microtiter (96-well) plate wells and incubated at 25°C for 24 hours. This served as inoculum for the secondary screen. For secondary screening; 100 μl of fungal culture was transferred into microtiter plate wells containing 100 μl minimal medium [37] supplemented with either 0, 6, 7, 8, 9 or 10% vv^-1^ ethanol (single well per treatment per transformant/wild type; see Figure S1B for plate layout). Plates were sealed with a lid and parafilm and incubated at 25°C for 96 hours after which fungal growth (OD_600nm_) was recorded. Data was normalised relative to the control (wild-type strain 11C). All datasets (0-10 vv^-1^ ethanol) were used for selecting transformants of interest. From the secondary screen, *F. oxysporum* strain 11C putative transformants demonstrating either ethanol sensitive or ethanol tolerant phenotypes relative to the wild-type strain 11C control, at concentrations ranging between 6-10% (vv^-1^) were selected for tertiary screening. This experiment was conducted once.

### Tertiary alcohol tolerance screen

In the tertiary screen, tolerance to both ethanol and butanol was assessed, with the goal of examining the potential for cross-correlation between ethanol and butanol tolerance. Conidial inoculum for the tertiary assessment was generated by individually inoculating each transformant into Mung bean broth [32] (100 ml conical flask) with three fungal plugs and incubating at 25°C and 150 rpm for 5 days. Inoculum was harvested by centrifugation and washed twice with sterile distilled water and resuspended in minimal medium [37] to a concentration of 10^6^ ml^-1^ conidia. Microtiter (96-well) plates were prepared with wells containing 100 μl minimal medium supplemented with either no alcohol, ethanol [2, 4, 6, 8 or 10% (vv^-1^)] or butanol (0.25, 0.5, 0.75, 1 and 1.25% (vv^-1^)] with a single treatment tested per 96-well plate (see Figure S1C for plate layout). Each plate was inoculated with 100 μl fungal conidia (10^6^ ml^-1^) (eight wells per treatment per wild type/transformant strain). Plates were sealed with a lid and parafilm and incubated at 25°C for 7 days. Fungal growth was measured using a spectrophotometer OD_600nm_ (Spectra Max 340 PC 96-well Plate Reader, Molecular Devices, USA), and percentage increase or decrease in growth relative to wild-type control was determined. For data analysis see Methods S1. This experimental screening was repeated three times.

### Phenotypic analysis of *F. oxysporum* 11C and Tr. 259

A series of phenotypic assays of *F. oxysporum* 11C and Tr. 259 were conducted to investigate the effect of increasing alcohol concentration on growth, temporal analysis of alcohol tolerance, the effect of alcohol tolerance stress on spore germination and biomass production (see Methods S1). All experiments were conducted either twice or three times. For data analysis see Methods S1.

### Genome walking and sequence analysis

 Genome walking was conducted on Tr. 259 to characterise the degree of genetic disruption underpinning the transformant’s observed phenotype (see Methods S1 & Table S1). 

### RT-PCR of putative hexose transporter (FOXG_09625) under alcohol stress

An experiment was designed to investigate the temporal accumulation of a putative hexose transporter under alcohol stress (Figure S2) (see Methods S1& Table S1). Each experiment was conducted either three times (no alcohol, ethanol) or twice (butanol) with three replicates per strain per treatment per time point. Real-time quantification of the target and housekeeping gene respectively were performed in separate reactions. The threshold cycle (*C*_T_) values obtained from RT-PCR were used to calculate the accumulation of the target gene (relative mRNA accumulation) relative to β-tubulin transcript by the 2^-ΔΔC^_T_ method, where ΔΔ*C*_T_ = (*C*_T_, target gene - C_T_, β-tubulin) [38]. Results were based on the average obtained for two replica RT-PCR reactions per sample. For data analysis see Methods S1.

### Phylogenetic analysis of putative sugar transporters in *F. oxysporum*

A phylogram of putative *F. oxysporum* strain 4287 sugar transporters was constructed using amino acid sequences from transcripts showing ≥30% homology to FOXG_09625 (See Methods S1). 

### Data analysis

The primary/secondary screen dataset was analysed using the Boxplot function in R (R v2.15.2 R Development Core Team, 2012) and a Box and Whisker plot was used to depict the distribution of transformant response across various treatments. The primary/secondary screen dataset was non-normally distributed as determined within Minitab (Minitab release © 16, 2011 Minitab Inc.). The significance between treatments at each time point (48 and 96 hours respectively) or ethanol treatments was analysed within the Statistical Package for the Social Sciences (SPSS 18, SPSS Inc.) using Kruskal-Wallis H-test for non-parametrical data. The significance between treatments and transformants amongst the 29 individuals selected from secondary screening was determined using one-way ANOVA as the data could be transformed to fit a normal distribution using Johnson transformation within Minitab. For tertiary screen analysis see Methods S1.

## Results

### ATMT library construction for primary alcohol screening

**Figure 1:**
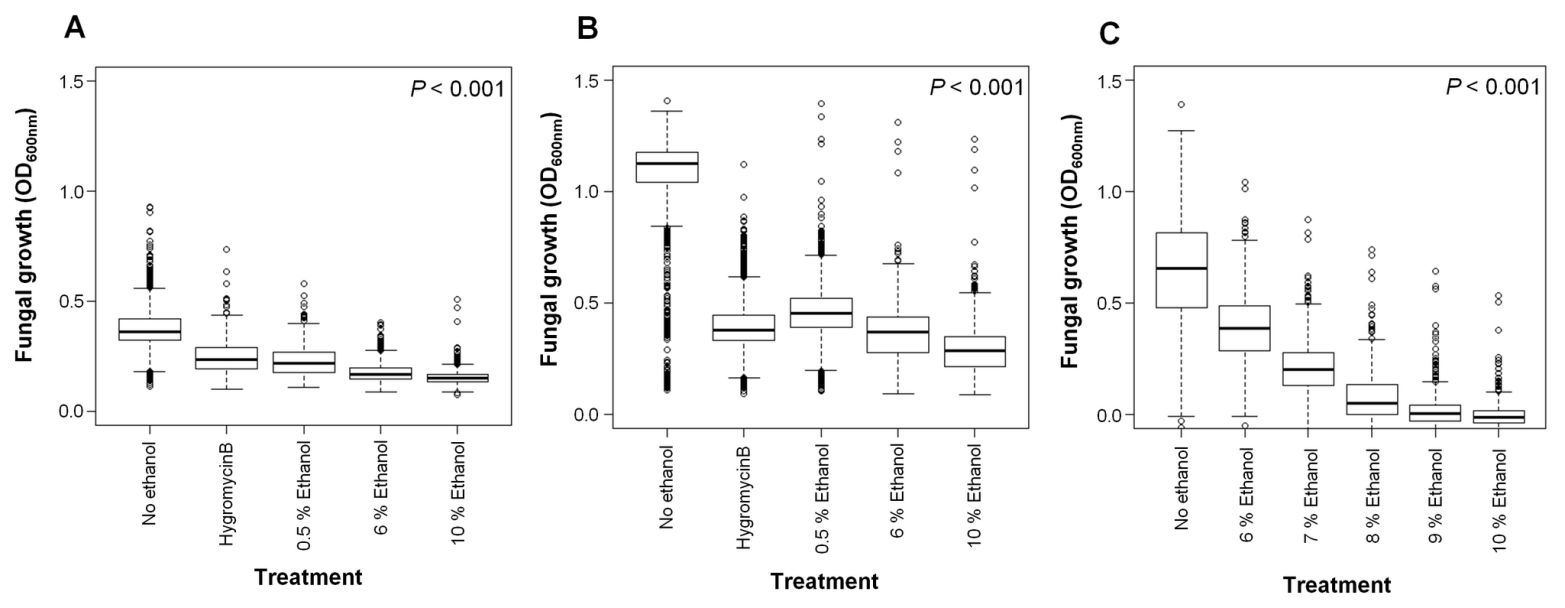
Primary and second – tier screening of putative *Fusarium* *oxysporum* strain 11C transformants. Box and Whisker plot depicting the distribution of putative *Fusarium* *oxysporum* strain 11C transformants (*n* =1,563) screened for altered ethanol tolerance during primary screening at 48 hours (**A**) and 96 hours (**B**) and transformants (*n* =402) screened for altered ethanol tolerance during secondary screening (**C**). Fungi were grown at 25°C in minimal medium [37] with five different treatments; no ethanol control; hygromycinB 60 µg ml^-1^ control; 0.5% (vv^-1^) ethanol; 6% (vv^-1^) ethanol; 10% (vv^-1^) ethanol) (Primary-tier) (**A**, **B**) or six treatments; no alcohol control; 6% (vv^-1^) ethanol; 7% (vv^-1^) ethanol; 8% (vv^-1^) ethanol; 9% (vv^-1^) ethanol; 10% (vv^-1^) ethanol (Second-tier) (**C**). Fungal growth (OD_600nm_) was measured after 96 hours using a spectrophotometer (Safire^2^, Tecan, Austria).

A transformation platform was established for *F. oxysporum* strain 11C to generate a library of gene disruption transformants (*n*=1,563) (Figure S3). The library was screened using a high throughput alcohol screen based on microtiter (96-well) plates to discriminate against low to high ethanol (0.5-10% (vv^-1^)) response levels measured across 48 and 96 hours (Figure 1A & 1B & Figure S3). Transformant selection for second – tier screening, as previously described [Methods], was based on recordings at 96 hours for which greater phenotypic variation was observed compared to 48 hours. An established threshold [Methods] led to the isolation of 182 putative transformants, which when purified (4 monoconidial cultures produced per putative transformant) yielded 443 viable monoconidial-derived cultures for PCR analysis and secondary alcohol screening.

### PCR analysis and secondary alcohol screening

Over 90% (402/443) of the monoconidial lines generated from primary transformants were PCR-positive for the *hph* transgene (Figure S4A). On re-screening the ethanol tolerance of this population, a significant difference between treatments was observed between the derived monoconidial lines (*P*<0.001) (Figure 1C). A total of 29 PCR-verified transformants indicating hypo-or hyper-ethanol growth phenotypes were selected for tertiary screening.

### Tertiary alcohol screening and transformant selection

**Figure 2:**
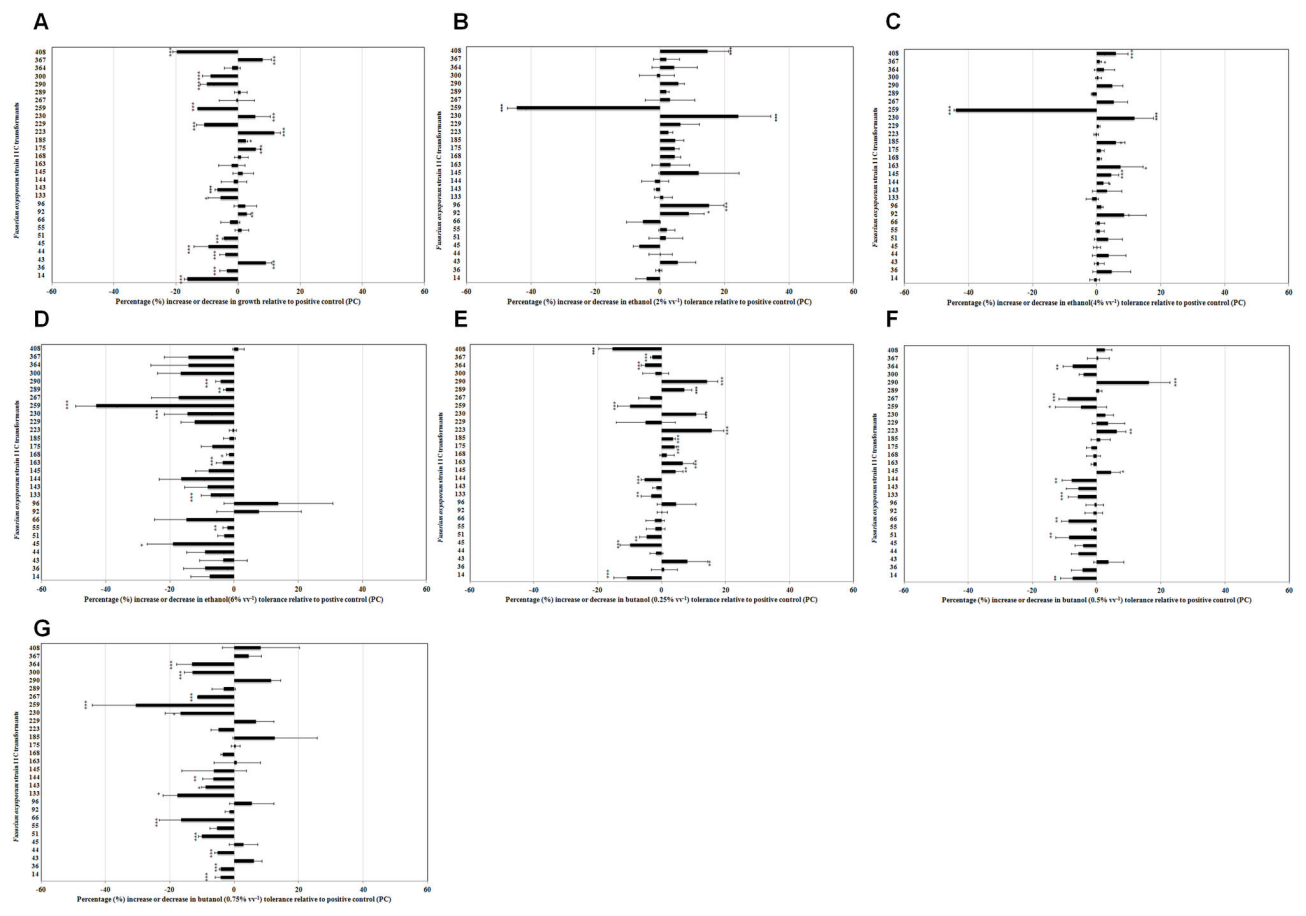
Tertiary screening of *Fusarium* *oxysporum* strain 11C transformants (*n*=29). Fungi were grown in minimal medium [37] supplemented with; no alcohol (**A**); 2% (vv^-1^) ethanol (ethyl alcohol; Sigma, UK) (**B**), 4% (vv^-1^) ethanol (**C**), 6% (vv^-1^) ethanol (**D**) or 0.25% (vv^-1^) butanol (*n*-butanol; Sigma, UK) (**E**), 0.5% (vv^-1^) butanol (**F**) or 0.75% (vv^-1^) butanol (**G**) and incubated at 25°C for 7 days. Percentage increase or decrease in fungal growth relative to the positive control (wild-type strain 11C) was determined. Results represent the mean of three independent experiments and bars indicate SEM. Transformants significantly different from the positive control (wild-type strain 11C) are highlighted with an asterisk (level of significance: <0.050 *, <0.01**, <0.001***).

Tertiary screening was used to assess tolerance of the 29 selected transformants to ethanol and cross-tolerance to butanol (Figure 2 & Table 1). A significant level of phenotypic diversity was recorded between strains across all treatments (*P*<0.001) (Figure 2). At 2 and 4% (vv^-1^) ethanol the highest level of hyper-or hypo-tolerance relative to parent strain 11C was observed for Tr. 230 and Tr. 259 (Figure 2B & 2C). At the highest ethanol concentration tested (6% vv^-1^), Tr. 259 continued to demonstrate a substantial hypo-tolerance to ethanol relative to the parental strain (-43.01%±6.40) (Figure 2D). A strong positive correlation was noted between ethanol and butanol treatments respectively (*r*=0.414; *n*=6, *P*=0.01). Similar to results recorded for ethanol, at the highest butanol concentration (0.75% vv^-1^) Tr. 259 (-30.61%±13.49) showed the highest hypo-butanol phenotype and Tr. 185 (12.57%±13.16) the most hyper-butanol phenotype relative to strain 11C (Figure 2G). 

**Table 1:** Summary of tertiary screening of *Fusarium* *oxysporum* strain 11C transformants to investigate the level of altered alcohol tolerance across a selected sub-population of individuals (*n*=29).

**Treat (vv^-1^)^a^**	**Population (%)^b^**	**Individuals (N)^c^**	**Hyper (N)^d^**	**Hypo (N)^e^**
Control 0	66	19	7	12
Ethanol 2	17	5	4	1
Ethanol 4	31	9	8	1
Ethanol 6	31	9	0	9
Butanol 0.25	62	18	9	9
Butanol 0.5	38	11	3	8
Butanol 0.75	45	13	0	13

a Positive control (no alcohol) or alcohol (ethanol or butanol) tested

b Percentage (%) population tested (*n*=29) showing hypo-hyper- phenotype relative to wild-type strain 11C (*P*<0.05)

c Total number of individuals showing hypo-hyper- phenotype relative to wild-type strain 11C (*P*<0.05)

d Total number of hyper-altered phenotypes (*P*<0.05)

e Total number of hypo-altered phenotypes (*P*<0.05)

**Figure 3:**
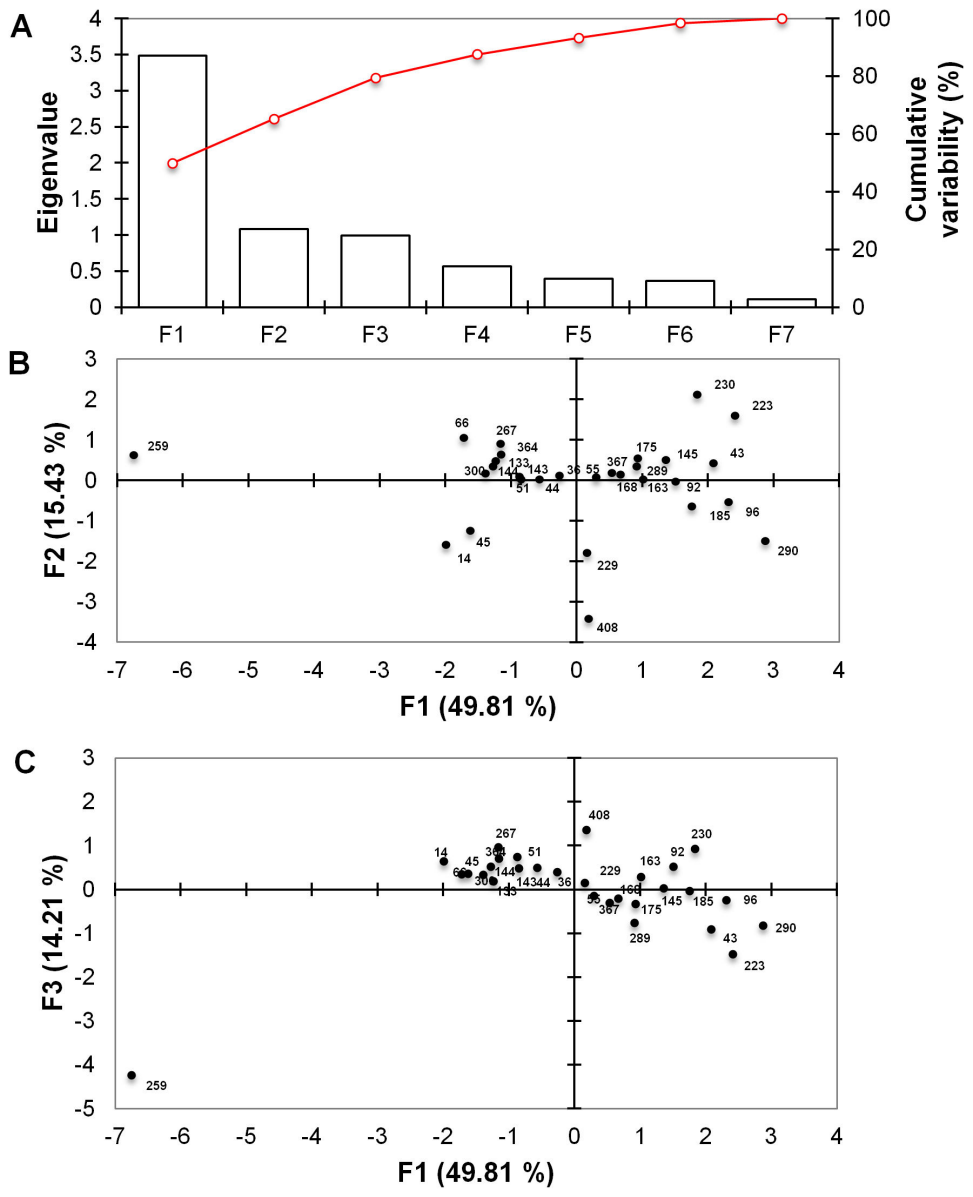
Principal component analysis (PCA) of tertiary screen. Principal component analysis (PCA) was used to reduce the dimension of the tertiary dataset comprising of several interrelated variables (no alcohol, ethanol and butanol treatments on all 29 transformants) whilst retaining the variation present within the dataset. The dataset was transformed into variables (F1-F7) through component analysis using XLSTAT (Addinsoft) v2013.1 software. Scree plot of PCA analysis with the first three Eigenvalues (F1-F3) corresponding to the highest percentage of variance (**A**) Observation plot (built on Scree plot) in the factor space according to the first and second Principal components F1 and F2 (**B**) Observation plot (built on Scree plot) in the factor space according to the first and second Principal components F1 and F3 (C).

 Southern blot analysis of a random sub-set of nine transformants using an *hph* probe indicated an average transgene copy number of 1.55 (Figure S4B). PCA analysis (Figure 3) highlighted the degree of phenotypic diversity among transformants and isolated Tr. 259 as a candidate strain of interest for further analysis. Southern analysis of this transformant suggests a single insertion event (data not shown). From the presented evidence, nine transformants were identified as candidate strains for future studies showing significantly (*P*<0.05) altered tolerance relative to the positive control (wild type strain 11C) to ethanol (respectively Tr. 14, 51,133,144 and 364), butanol (respectively Tr. 92, 230 and 408) or significantly(*P*<0.05) for both alcohols, Tr. 259.

### Phenotypic analysis of Tr. 259

The effect of alcohol stress on temporal growth, spore germination and biomass production was investigated to confirm the hypo-tolerant phenotype of Tr. 259 observed during tertiary screening. The effect of increasing alcohol concentration on strain growth was firstly assessed (Figure S5). Growth of both Tr. 259 and 11C decreased with increasing ethanol (*r*≥-0.967, *n*=5, *P*≤0.02) and butanol (*r*≥-0.929, *n*=8, *P*≤0.01) concentration whereas 11C showed significantly better growth than Tr. 259 up to 6% (vv^-1^) ethanol and 1% (vv^-1^) butanol (*P*<0.05) (Figure S5). At 4% (vv^-1^) ethanol and 0.75% (vv^-1^) butanol a substantial difference in growth (≥42%) was recorded between Tr. 259 and 11C prompting the selection of these concentrations for further studies (Figure S5). 

**Figure 4:**
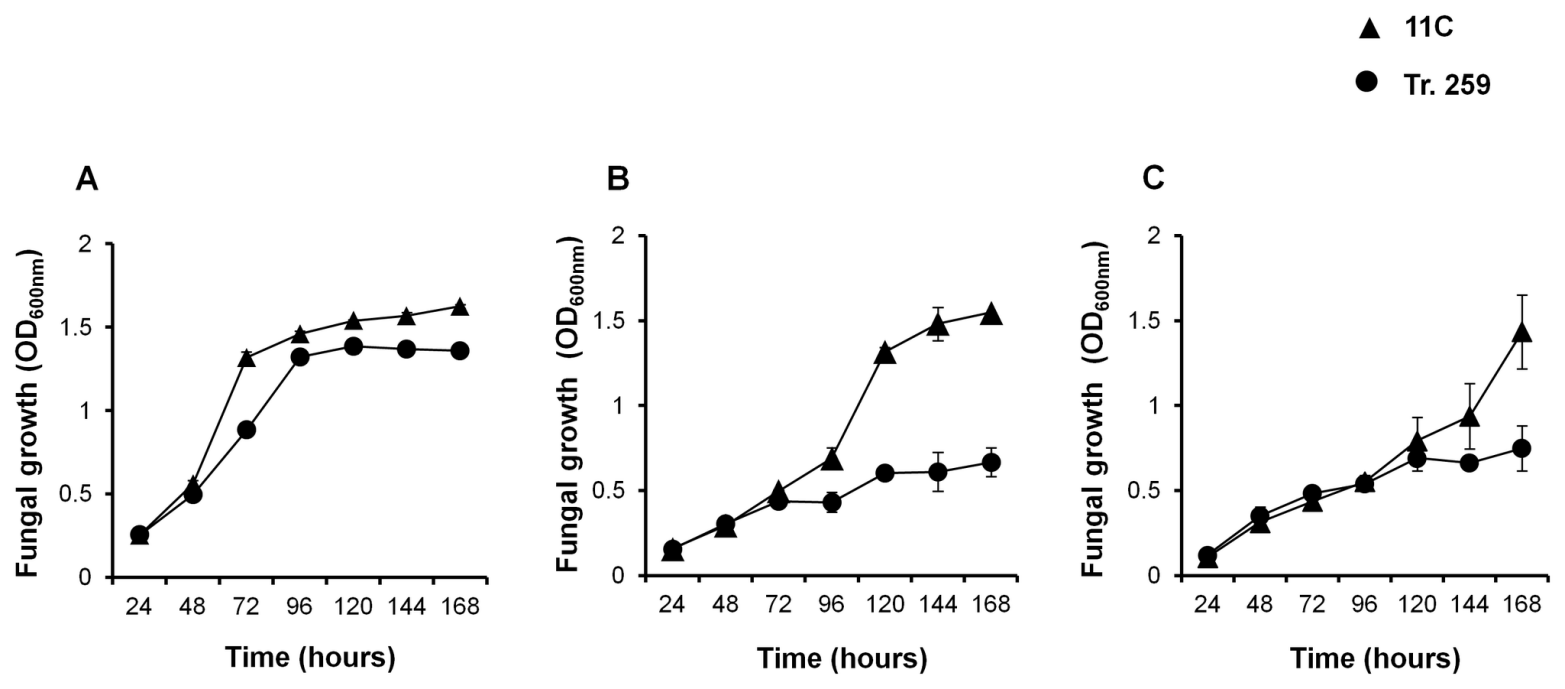
Temporal analysis of alcohol tolerance in *F. oxysporum* Fungal conidial inoculum was produced in Mung bean broth [32] and resuspended in minimal medium [37] at a concentration of 10^6^ ml^-1^. A volume 100 µl conidia (10^6^ ml^-1^) was added to microtiter (96-well; Sarstedt, Germany) plates with either no alcohol (**A**), ethanol (Ethyl absolute, Sigma, UK) at a concentration of 4% (vv^-1^) (**B**) or butanol (*n*-Butanol Butyl alcohol, Sigma, UK) at a concentration of 0.75% (vv^-1^) (**C**). Fungi were maintained at 25°C for 168 hours and growth (OD_600nm_) was measured every 24 hours. Fungal growth (OD_600nm_) was measured using a spectrophotometer (Spectra Max 340 PC 96-well plate reader, Molecular Devices, USA). Bars indicate SEM. Growth of Tr. 259 significantly different from 11C is highlighted with an asterisk (level of significance: *<0.05, ** < 0.01).

In the absence of alcohol, both Tr. 259 and 11C exhibited similar mycelial growth and spore germination rates (Figure 4A & Figure S6D). In contrast, 11C achieved better mycelial growth with either 4% ethanol or 0.75% butanol present in the medium, as compared to Tr. 259 (Figure 4B & 4C). Both 4% ethanol and 0.75% butanol severely impacted spore germination, however 11C achieved significantly greater spore germination than Tr. 259 (*P*≤0.01) (Figure S6B-C & S6E-F).

### Genomic analysis of Tr. 259

Genome walking was conducted to determine the potential genomic region associated with Tr. 259’s hypo-tolerant phenotype (Figure S7). This resulted in the cloning of a 1031nt sequence external to the T-DNA right border (RB) (sequence analysis confirmed the amplicon was flanked by the universal and RB specific primer as expected; Figure S8A). Further analysis did not result in the recovery of full-length T-DNA in the identified coding region, indicating partial T-DNA integration. *In silico* analysis of the recovered coding sequence showed the highest homology in the ORF of a putative sugar transporter gene FOXG_09625 in *F. oxysporum* f.sp.*lycospersici* (strain 4287) at both the protein (68% identity) and nucleotide (83% identity) level respectively (Figure S8B & S8C). 

 FOXG_09625 showed homology (percent identity ≥ 30%) with 30 different proteins all containing MFS and sugar transporter domains respectively, encoded within the genome of *F. oxysporum* f.sp.*lycospersici* (strain 4287) (FGCD). Five of these proteins were annotated as hexose transporters (FOXG_02491, FOXG_09722, FOXG_05876, FOXG_13263, FOXG_014666) including FOXG_09625, and the remaining as hypothetical proteins. Comparison of these proteins showed considerable sequence diversity indicating potentially diverse roles of these proteins in *F. oxysporum* (Figure S9A). In total, the genome of *F. oxysporum* f.sp. *lycospersici* (strain 4287) encodes 13 proteins annotated as hexose transporters (Figure S9B).

**Figure 5:**
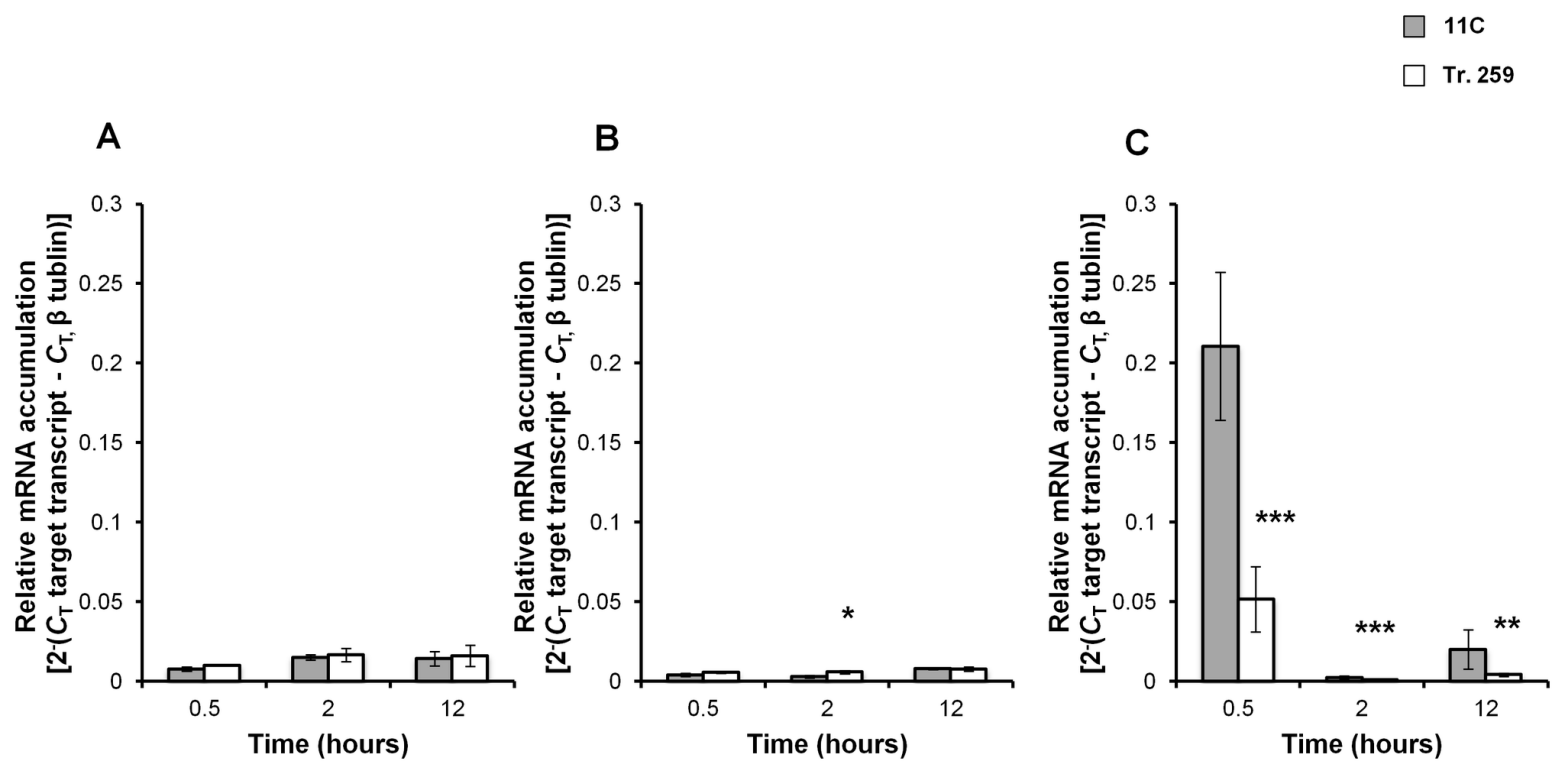
RT-PCR analysis of putative hexose transporter under alcohol stress. Temporal analysis of putative hexose transporter transcript (FOXG_09625) during shake flask growth of *Fusarium* *oxysporum* strain 11C and transformant Tr. 259 under normal conditions (**A**), ethanol stress (**B**) and butanol stress (**C**). *F*. *oxysporum* (2ml 10^6^ ml^-1^) was aerobically cultured in Erlenmeyer flasks (100 ml) in 48 ml of minimal medium [37] for 12 hours shaking at 150 rpm at 25°C. At 12 hours, flasks were amended with 2ml of water, ethanol or butanol to a final concentration of 0%, 4% and 0.75% (vv^-1^), respectively. RT-PCR was conducted on RNA samples harvested at 0.5, 2, 12 hours respectively post – inoculation. Putative HXT accumulation was quantified relative to the housekeeping gene β-tubulin (FOXG_06228) [38] Results are based on three experiments (no alcohol, ethanol) or two experiments (butanol), each with three replicates per time point per strain tested. RT-PCR was conducted twice on each experiment. Bars indicate SEM. Tr. 259 FOXG_09625 mRNA accumulation significantly different from strain 11C is highlighted with an asterisk (level of significance: *<0.05, **≤ 0.01, ***< 0.001).

 RT-PCR analysis was used to analyse FOXG_09625 transcript levels in strain 11C compared to Tr. 259, following short-term (0.5-12 hours) cultivation of germinated spores (pre-cultured for 12 hours) in the absence or presence of alcohol (Figure 5). In the absence of alcohol, basal expression was low in both 11C and Tr. 259 (*P*>0.05). (Figure 5A). Reduced transcript levels were recorded for both strains under ethanol stress compared to normal conditions from 0.5 to 12 hours with a significant difference between strains at 2 hours (*P*<0.01) (Figure 5B). In the presence of butanol, a significant difference between Tr. 259 and 11C was recorded for all time points tested (*P*<0.001) with transcript levels highest at 0.5 hours (Figure 5C).

## Discussion

This study has shown that ATMT is an effective tool to generate phenotypic diversity in response to alcohol stress within *F. oxysporum* strain 11C. Although the generation of fungal insertion libraries is well documented, the integration of a three-tier alcohol screen into an ATMT platform is novel, and led to the generation and isolation of phenotypically diverse transformants from the library, indicating genotype-dependent variation and a functional ATMT system.

 Ethanol tolerance of strain 11C (>6% (vv^-1^) ethanol) was high compared to *Candida albicans*, which like *F. oxysporum* can ferment glucose to ethanol but can only tolerate 1% (vv^-1^) ethanol stress [39]. In contrast, yeast can tolerate high ethanol concentrations (>15% (vv^-1^)), however at lower concentrations (4-6% (vv^-1^)) a 50% reduction in yeast’s growth has been reported [40]. Unsurprisingly, strain 11C could not tolerate equivalent butanol concentrations as a barrier between 1-2% (vv^-1^) exists for most microbes with the exception of some *Lactobacillus* strains (3% (vv^-1^)) [41] and *Bacillus subtilis* (5% (vv^-1^)) [42].

 Large-scale screening of filamentous fungi can be challenging as manipulating fungal material (e.g. mycelium) can interfere with pipette-aided transfer between plates [43]. Fungal growth rates or hyphal growth patterns can vary greatly among isolates with some strains growing poorly in low volume space-limited microplates. Regardless, microplate-based assays facilitate the screening of large populations: a significant advantage over the labour-intensive regime of individual shake flasks or tubes [43]. Screening of *F. oxysporum* ATMT-derived transformants for alcohol tolerance has not yet been described. In other ascomycetous fungi notably the rice blast fungus *Magnaporthe grisea*, large-scale ATMT mutagenesis coupled with phenotypic plate-based screens has been used in pathogenicity related studies [44,45].

 The three-tier screening system was designed to specifically discriminate either alcohol sensitive or tolerant phenotypes. Integration of the primary screen into the ATMT protocol provided a course but effective method to instigate a logistically feasible sorting of the library, thereby avoiding the laborious and time-consuming task of purifying thousands of putative transformants prior to screening. Non-purified, putative transformants underwent primary screening in contrast to the secondary screen, which used single-spored *hph*-PCR positive transformants. It cannot be ruled out that false negatives were lost to the analysis during these two stages. These primary rounds of screening were based on single reads to accelerate the phenotyping process and isolate transformants of interest for further analysis using an intensive confirmatory tertiary screen. This latter screen highlighted transformant Tr. 259 which underwent phenotypic and genomic analysis to confirm its hypo-alcohol tolerant phenotype and the ATMT-based disruption of a coding region showing homology to a putative sugar transporter (FOXG_09625) in *F. oxysporum* f.sp *lycospersici* (strain 4287). Southern hybridisation suggested a single insertion site within Tr. 259 with genome walking indicating partial T-DNA integration but it cannot be ruled out that additional T-DNA insertion events have also occurred through the genome of Tr. 259. 

 Eukaryotic transporters are best characterised in the yeast 20-member family of major hexose transporter genes (HXT1-4, HXT6-7, HXT8-17) which includes two sensor-coding genes (SNF3, RGT2) and the GAL2 gene [46]. In contrast to yeasts (*S. cerevisiae*, *Pichia stipitis*, *Schizosaccharomyces pombe*, *Kluveromyces lactis*), few sugar transporters have been identified and characterised in filamentous fungi [47,48]. The genome of *F. oxysporum* f.sp *lycospersici* (strain 4287) has at least 13 putative hexose transporters including FOXG_09625, similar to *Aspergillus nidulans* reported to have 17 [47]. RT-PCR analysis indicated FOXG_09625 was differentially expressed in Tr. 259 compared to WT during alcohol-induced stress and it would be interesting to investigate by Western blotting whether FOXG_09625 is also differentially expressed at the protein level. In yeast, the closest protein homologue of FOXG_09625 is HXT13, which is induced in the presence of non-fermentable carbon sources (e.g. alcohols), low sugar levels and repressed under high sugar concentrations [49]. It was hypothesised that FOXG_09625 would be poorly expressed under normal conditions and highly expressed in the presence of ethanol or butanol. Whilst this hypothesis was not observed under ethanol stress, results recorded for butanol were as expected. For yeast, the association of hexose transport and glycolysis associated genes with higher expression levels under 4% (vv^-1^) ethanol stress led to the proposal that the cell enters a pseudo-starvation state during ethanol stress, whereby sugar-based nutrients are no longer accessible to the cell despite being present in the culture medium [50,51]. In this study, the highest FOXG_09625 transcript levels were recorded under butanol stress (0.5 hours) suggesting that cells may have immediately entered a pseudo-starvation state in response to the increased toxicity of butanol, possibly inhibiting sugar up-take. In yeast, increased expression of hexose transporters and glycolysis genes improve both rapid production and consumption of ethanol [52].

 A previous study [53] identified two sugar transporters overexpressed in strain 11C during CBP. In the analysis of additional transformants we would expect to identify an overlap with the SSH library for genes holding dual function i.e. relating to both production and tolerance. However, ethanol stress studies to date have identified genes belonging to diverse functional categories hence the identification of novel genes could also be anticipated. Interestingly, FOXG_09625 showed 29% protein homology to the SSH – identified hexose transporter and no homology to the high affinity glucose transporter indicating potentially different roles for all three genes in either tolerance or production. Yet, a recent study [54] has indicated FOXG_09625 is associated (directly/indirectly) with ethanol production since mutants overexpressing a hexose transporter resulted in increased ethanol yield coupled with compensatory changes in the expression of other transporters notably the up-regulation of FOXG_09625. Investigations, including overexpression/knockout studies of FOXG_09625, while not possible due to a limitation in resources for this study will be required to fully elucidate the complexity of this gene interaction with alcohol and determine the degree to which over-expression/silencing of FOXG_09625 could improve consolidated bioprocessing yield.

 Filamentous fungal transporters appear to hold diverse functional roles notably in plant-fungi symbiosis [55-57] pathogenicity [58,59], and ethanol production and tolerance [48,50,60-62]. In regards to tolerance, transcriptional studies have identified alcohol responsive genes associated with sugar transport [50,63,64]. This is not surprising since the rate of sugar transport is limited by accumulation of alcohol in the fermentation broth, which can become a significant stress during fermentation resulting in a ‘survival versus production’ conundrum for the producing microbe [65]. While major advances have been made investigating alcohol tolerance in model systems (yeast, *E. coli*), a knowledge deficit exists for filamentous fungi [66-68]. A better understanding of the molecular basis of alcohol stress and tolerance in such fungi is important if enhanced tolerance and production is to be achieved. Previous work [54] coupled with this study point towards sugar transporters as key targets for improving fungal-enabled CBP.

 In conclusion, the work completed in this study represents a first step in the investigation of alcohol tolerance in *F. oxysporum* via ATMT, which has led to the generation of a collection of transformants, which are now available to the research community for future studies. This is all the more relevant in light of the recent advances and application of RNA sequencing to elucidate complex biological processes at the transcript level in an unlimited array of organisms [69]. On the basis of the results presented here, ATMT can be exploited as a tool to generate diverse alcohol tolerant phenotypes in the CBP agent *F. oxysporum*.

## Supporting Information

Figure S1 **Microplate (96-well; Sarstedt, Germany) layout for three-tier screening.** *Fusarium oxysporum* wild-type strain 11C (WT) and putative transformants were inoculated into columns (1-12) and screened across five treatments: T1; no ethanol, T2; hygryomcinB (60 μg ml^-1^; Sigma, UK), T3; 0.5% (vv^-1^) ethanol (ethyl alcohol; Sigma, UK), T4; 6% (vv^-1^) ethanol T5; 10% (vv^-1^) ethanol with treatments positioned in rows (A-F) (Primary-tier) (**A**) or into rows (A-H) and then transferred and inoculated into corresponding rows in a second microplate and screened across six treatments: T1; no ethanol, T2; 6% (vv^-1^) ethanol (ethyl alcohol; Sigma, UK), T3; 7% (vv^-1^) ethanol T4; 8% (vv^-1^) ethanol, T5; 9% (vv^-1^) ethanol, T6; 10% (vv^-1^) ethanol (Second-tier) (**B**) or into columns (1-12) with eight replicates per strain tested (column A-H) and screened across a single treatment including media control (MC) per plate: either no alcohol, 2% (vv^-1^) ethanol (ethyl alcohol; Sigma, UK), 2% (vv^-1^) ethanol, 4% (vv^-1^) ethanol, 6% (vv^-1^) ethanol or 0.25% (vv^-1^) butanol, 0.5% (vv^-1^) butanol and 0.75% (vv^-1^) butanol (**C**). Grey shading indicates microplate well not used during experiment.
(TIF)

Figure S2 **Experimental design flow chart to investigate alcohol-induced stress in *Fusarium oxysporum* strain 11C and transformant Tr. 259.**
(TIF)

Figure S3 **Generating and screening *Fusarium oxysporum* strain 11C transformants.** Inoculation of *Fusarium oxysporum* strain 11C into Mung bean broth [32] for 5 days at 25°C and collection of conidia by filtration through sterile cheesecloth, washing twice with sterile distilled water and adjusting spore concentration to 10^4^ conidia per ml (**A**) Inoculation of *Agrobacterium* *tumefaciens* strain AGL-1 to minimal medium (MM) [34] supplemented with Kanamycin (50 μg ml^-1^) for 2 days at 28°C (OD_600nm_ 0.4-0.6) followed by dilution to OD_600nm_ of 0.15 in induction medium (IM) [34] supplemented with 200 μM acetosyringone (AS) and incubation for 6 hours at 28°C (**B**) Co-cultivation of an equal volume of bacterial and fungal cells for 30 minutes at 28°C in liquid co-cultivation medium [34] followed by spreading 100 μl mix onto a UV-sterilised cellulose filter membrane placed on solid co-cultivation medium on large Petri plates (150x20mm) for 2 days at 25°C where if transformation is successful, T-DNA is transferred from AGL-1 to *F. oxysporum* strain 11C and randomly integration into the fungal genome results (**C**) Transfer of cellulose filter membrane to modified selection medium (SM) on large Petri plates (150x20mm) supplemented with 60 hygromycinB for 7-9 days at 25°C followed by isolation of hygromycinB (60 μg ml^-1^) resistant putatively transformed colonies into microtiter (96-well) plates with minimal medium [37] supplemented with hygromycinB (60 μg ml^-1^) for 3 days at 25°C (**D**) Transfer of putative transformants to microtiter (96-well) plate with minimal medium supplemented with ethanol and butanol selection for primary alcohol tolerance screening followed by purification and PCR prior to two additional rounds of screening (2° and 3°) and selection of candidates for future analysis (**E**).
(TIF)

Figure S4 **Molecular analysis of *Fusarium oxysporum* strain 11C transformants.** PCR was used to determine if fungal transformant genomic DNA (gDNA) extracts contained *hph* gene present in pSK1019. Lanes 2-6 represent gDNA from transformants, 7; PC – positive control (plasmid DNA pSKI019), 8; NTC – no template control (water), 9; WT - gDNA from wild-type strain 11C. Arrow indicates PCR product size (576 bp) based on 100bp ladder (NEB, UK) (Lane 1) (**A**). Southern blot analysis was used to confirm transgene integration in *Eco*R1 digested fungal gDNA and to determine copy number, using a 741nt fragment of the hygromycin phosphotransferase gene (*hph*) as a probe. Lanes 1-11 represent DNA digested with *Eco*R1; 1, gDNA from wild type fungal strain 11C, 2-10, gDNA from strain 11C transformants Tr. 144, Tr. 175, Tr. 230, Tr. 51, Tr. 43, Tr. 133, Tr. 145, Tr. 55 and Tr. 168, 11, plasmid DNA from pSK1019. Red dots indicate bands and arrows indicate molecular size (Kb) based on 1 Kb DNA ladder (Solis BioDyne, Estonia) (**B**).
(TIF)

Figure S5 **Effect of increasing alcohol concentration on *Fusarium oxysporum* growth.** Fungal conidial inoculum was produced in Mung bean broth [32] and resuspended in minimal medium [37] at a concentration of 10^6^ ml^-1^. A volume 100 µl conidia (10^6^ ml^-1^) was added to microtiter (96-well; Sarstedt, Germany) plates supplemented with either ethanol (Ethyl Absolute, Sigma, UK) (**A**) or butanol (*n*-Butanol Butyl alcohol, Sigma, UK) (B). Fungi were maintained at 25°C for 7 days. Fungal growth (OD_600nm_) was measured using a spectrophotometer (Spectra Max 340 PC 96-well plate reader, Molecular Devices, USA). Bars indicate SEM. Growth of Tr. 259 significantly different from 11C is highlighted with an asterisk (level of significance: *<0.05, ** < 0.01).
(TIF)

Figure S6 **Effect of alcohol stress on spore germination and biomass production in *F. oxysporum*.** Spore germination of *F. oxysporum* strain 11C and transformant Tr. 259 in the presence of no alcohol (**A**) 4% (vv^-1^) ethanol (**B**) and 0.75% (vv^-1^) butanol (**C**); fungal conidial inoculum was produced in Mung bean broth [32] and resuspended in minimal medium [37] at a concentration of 10^6^ ml^-1^. Fungi were maintained at 25°C for 24 hours shaking at 150 rpm and percentage germination was measured every 2 hours. Bars indicate SEM. Biomass production of *F. oxysporum* strain 11C and transformant Tr. 259 post – treatment with either; no alcohol (**D**) 4% (vv^-1^) ethanol (**E**) or 0.75% (vv^-1^) butanol (**F**). Minimal medium (48 ml) was inoculated with fungal conidia (2ml of 10^6^ ml^-1^) and flasks were incubated at 25°C, 150 rpm. After 12 hours, flasks were amended with 2ml of water, ethanol or butanol to a final concentration of 0, 4 and 0.75% (vv^-1^), respectively. Fungal material was collected at 72, 120 and 168 hours post treatment. Bars indicate SEM. Spore germination or biomass production of Tr. 259 significantly different from 11C is highlighted with an asterisk (level of significance: *<0.05, ** < 0.01).
(TIF)

Figure S7 **Genome walking in *F. oxysporum* 11C transformant Tr. 259.** Genome walking analysis using APA^TM^ Gold Genome Walking Kit (Bio S&T, Canada) to walk the right border (RB) of pSK1019. Lanes represent: 1 - 4; degenerate random tagging (DRT) primers A, B, C and D respectively with target specific primer (GSP) for RB of pSK1019 amplified with 500 ng of fungal genomic DNA from *Fusarium oxysporum* strain 11C transformant Tr. 259. Arrow indicates PCR fragment sizes (bp) on Hyperladder™ III (Bioline, UK) (Lane 5). Red dots indicate gel excised bands for subsequent cloning (**A**). Digestion of plasmid DNA from pGEMT ® easy (Promega, UK) cloned PCR products to verify correct fragment insert size. Plasmid DNA (500 ng) was digested in 20 µl reaction and incubated at 37°C for 2 hours. Lanes represent: 1, Hyperladder™ III (Bioline, UK), 2 - 5, bands excised from Figure S7A highlighted by red dots; 6, blue colony (negative control) (**B**). PCR analysis of cloned PCR products to verify presence of target specific primer (GSP) used in genome walking prior to sequencing. Lanes represent: Lanes represent: 1, Hyperladder™ III (Bioline, UK), 2 - 5, bands excised from Figure S7A highlighted by red dots; 6, blue colony (negative control); 7, no plasmid DNA (negative control) (**C**).
(TIF)

Figure S8 ***Insilico* analysis of identified coding sequence obtained via genome walking in Tr. 259.** Sequence analysis of cloned PCR product obtained via genome walking within *Fusarium oxysporum* strain 11C transformant Tr. 259. Walking was from the right border (RB) of the transformation vector pSK1019 using the APA^TM^ Gold Genome Walking Kit (Bio S&T, Canada), a universal kit primer (UAP-2) (red) and the gene-specific (GSP) primer (blue) (3’-5’) (**A**). Identified coding sequence was analysed against the *F. oxysporum* f.sp.*lycospersici* (strain 4287) genome (FCGD; (http://www.broadinstitute.org/annotation/genome/fusarium_group)) using Blastn (**B**) and Blastx (**C**).
(TIF)

Figure S9 **Phylogenetic analysis of putative sugar transporters in *Fusarium oxysporum*.** Homology (≥30%) of FOXG_09625 to putative sugar transporters in *F. oxysporum* 4287 (**A**). Comparison of putative hexose transporters in *F. oxysporum* 4287 compared to *Saccharomyces cerevisiae* and putative sugar transporters showing (≥30%) homology to FOXG_09625 in *Aspergillus nidulans, Tricoderma reesei, Mucor* circinelloides*, Neurospora crassa* (**B**). Amino acid sequences were obtained from FGCD (http://www.broadinstitute.org/annotation/genome/fusarium_group) SGD (http://www.yeastgenome.org) and NCBI (http://www.ncbi.nlm.nih.gov). All protein sequences showed sugar transporter domains and were aligned using ClustalW. A phylogram was constructed using the Neighbor-Joining method within Phylogeny.fr software. Numbers on internal nodes represent bootstrap values (100 re-sampling). Arrow indicates putative hexose transporter (FOXG_09625) used in RT-PCR analysis. Asterisk indicates transcripts annotated as hexose transporters in *F. oxysporum* 4287 (FGCD).
(TIF)

Table S1 **Primers used in this study for genome walking and real-time PCR.**
(DOC)

Methods S1 **Expanded description of methods.**
(DOCX)

References S1 (DOCX)
