# Supplementary figures and images for: Generating Phenotypic Diversity in a Fungal Biocatalyst to Investigate Alcohol Stress Tolerance Encountered during Microbial Cellulosic Biofuel Production
Source: PLoS One. 2013 Oct 16;8(10):e77501. doi: 10.1371/journal.pone.0077501 (PMC3797763; doi:10.1371/journal.pone.0077501)

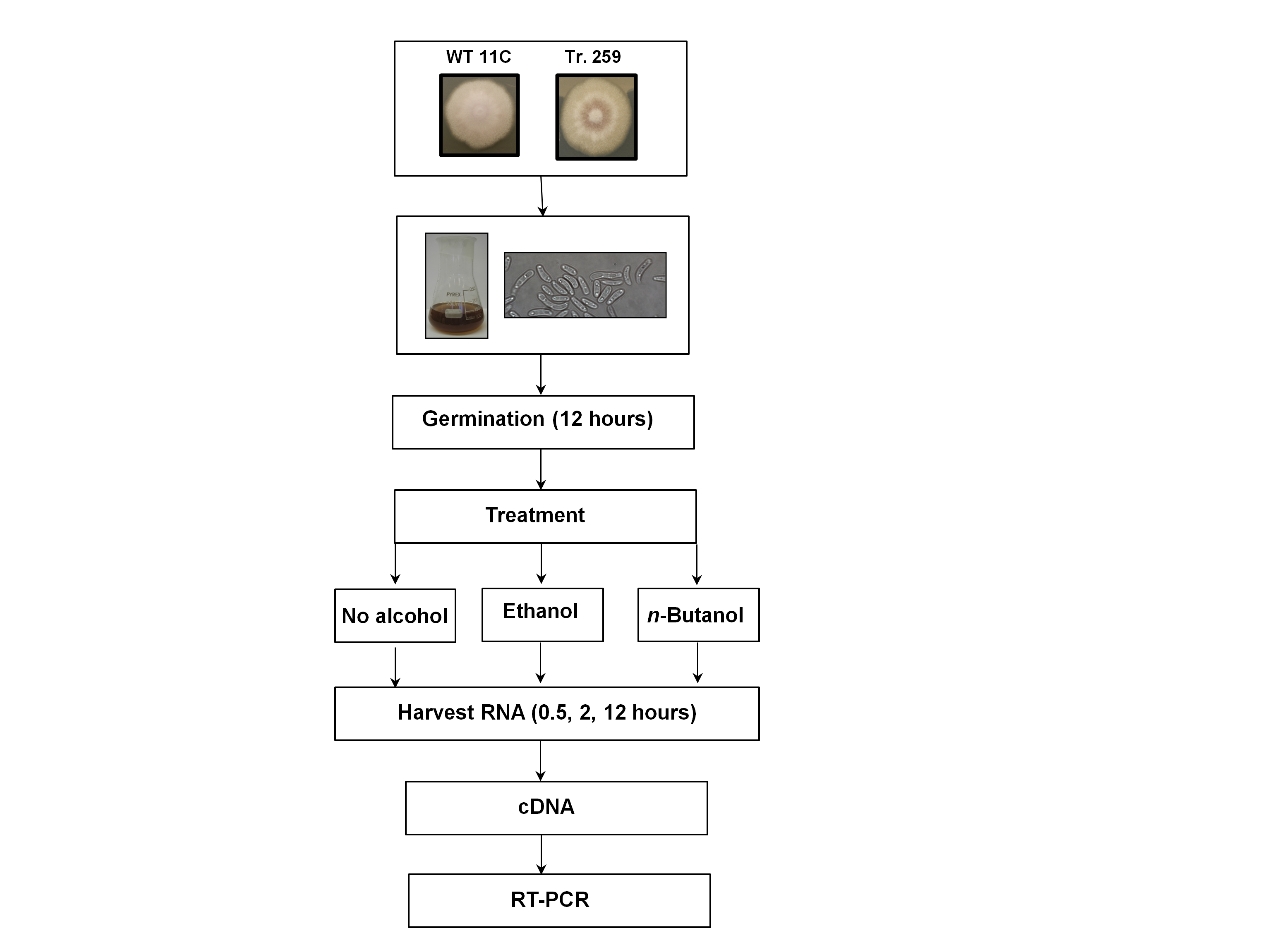

Supplement: Figure S2 — Experimental design flow chart to investigate alcohol-induced stress in Fusarium oxysporum strain 11C and transformant Tr. 259. (TIF) [file pone.0077501.s002.tif]

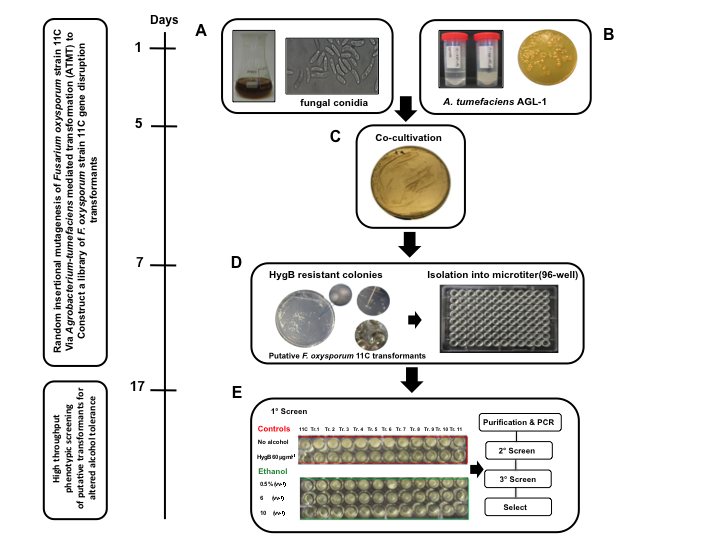

Supplement: Figure S3 — Generating and screening Fusarium oxysporum strain 11C transformants. Inoculation of Fusarium oxysporum strain 11C into Mung bean broth [32] for 5 days at 25°C and collection of conidia by filtration through sterile cheesecloth, washing twice with sterile distilled water and adjusting spore concentration to 104 conidia per ml (A) Inoculation of Agrobacterium tumefaciens strain AGL-1 to minimal medium (MM) [34] supplemented with Kanamycin (50 μg ml-1) for 2 days at 28°C (OD600nm 0.4-0.6) followed by dilution to OD600nm of 0.15 in induction medium (IM) [34] supplemented with 200 μM acetosyringone (AS) and incubation for 6 hours at 28°C (B) Co-cultivation of an equal volume of bacterial and fungal cells for 30 minutes at 28°C in liquid co-cultivation medium [34] followed by spreading 100 μl mix onto a UV-sterilised cellulose filter membrane placed on solid co-cultivation medium on large Petri plates (150x20mm) for 2 days at 25°C where if transformation is successful, T-DNA is transferred from AGL-1 to F. oxysporum strain 11C and randomly integration into the fungal genome results (C) Transfer of cellulose filter membrane to modified selection medium (SM) on large Petri plates (150x20mm) supplemented with 60 hygromycinB for 7-9 days at 25°C followed by isolation of hygromycinB (60 μg ml-1) resistant putatively transformed colonies into microtiter (96-well) plates with minimal medium [37] supplemented with hygromycinB (60 μg ml-1) for 3 days at 25°C (D) Transfer of putative transformants to microtiter (96-well) plate with minimal medium supplemented with ethanol and butanol selection for primary alcohol tolerance screening followed by purification and PCR prior to two additional rounds of screening (2° and 3°) and selection of candidates for future analysis (E). (TIF) [file pone.0077501.s003.tif]

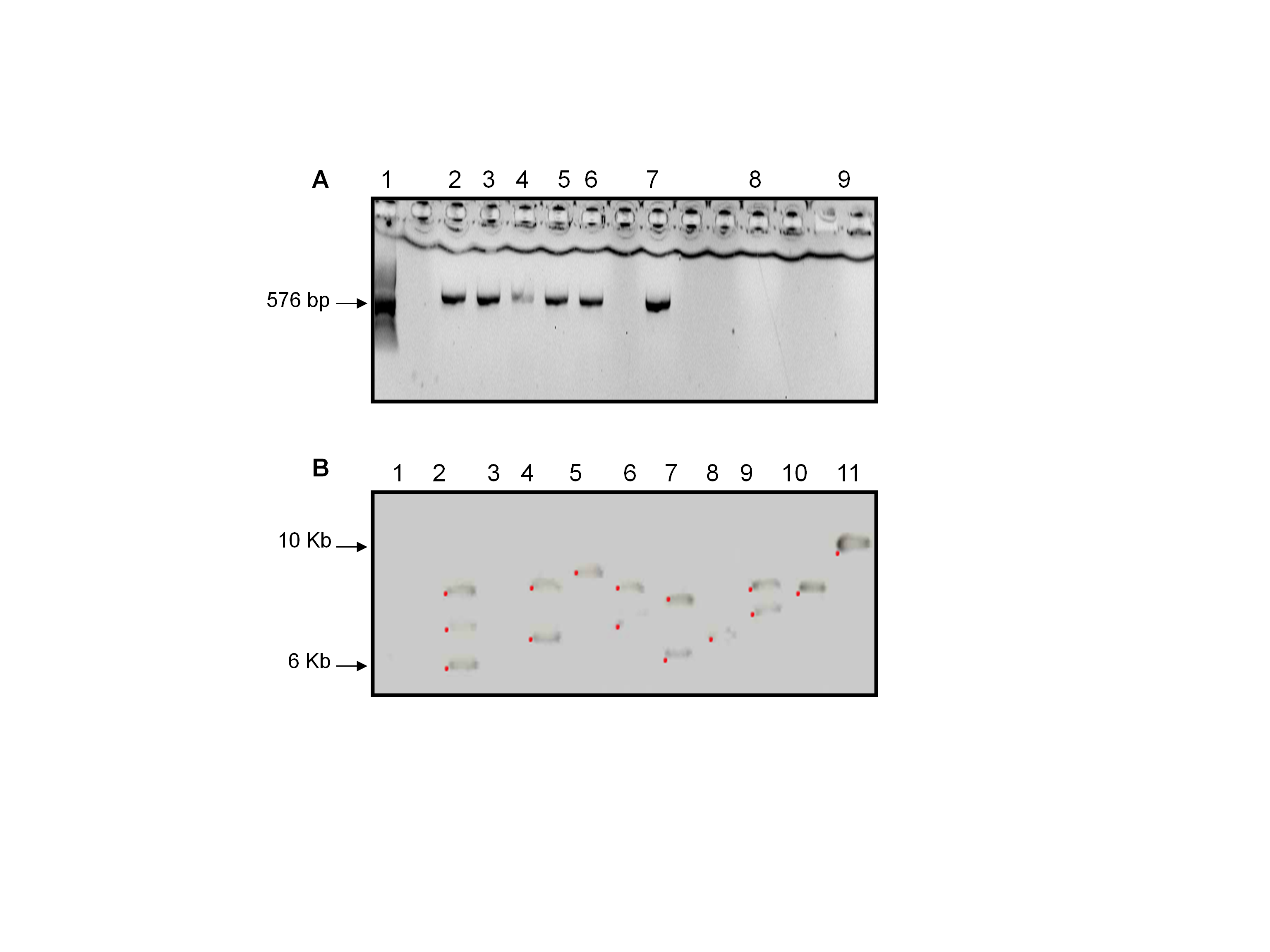

Supplement: Figure S4 — Molecular analysis of Fusarium oxysporum strain 11C transformants. PCR was used to determine if fungal transformant genomic DNA (gDNA) extracts contained hph gene present in pSK1019. Lanes 2-6 represent gDNA from transformants, 7; PC – positive control (plasmid DNA pSKI019), 8; NTC – no template control (water), 9; WT - gDNA from wild-type strain 11C. Arrow indicates PCR product size (576 bp) based on 100bp ladder (NEB, UK) (Lane 1) (A). Southern blot analysis was used to confirm transgene integration in EcoR1 digested fungal gDNA and to determine copy number, using a 741nt fragment of the hygromycin phosphotransferase gene (hph) as a probe. Lanes 1-11 represent DNA digested with EcoR1; 1, gDNA from wild type fungal strain 11C, 2-10, gDNA from strain 11C transformants Tr. 144, Tr. 175, Tr. 230, Tr. 51, Tr. 43, Tr. 133, Tr. 145, Tr. 55 and Tr. 168, 11, plasmid DNA from pSK1019. Red dots indicate bands and arrows indicate molecular size (Kb) based on 1 Kb DNA ladder (Solis BioDyne, Estonia) (B). (TIF) [file pone.0077501.s004.tif]

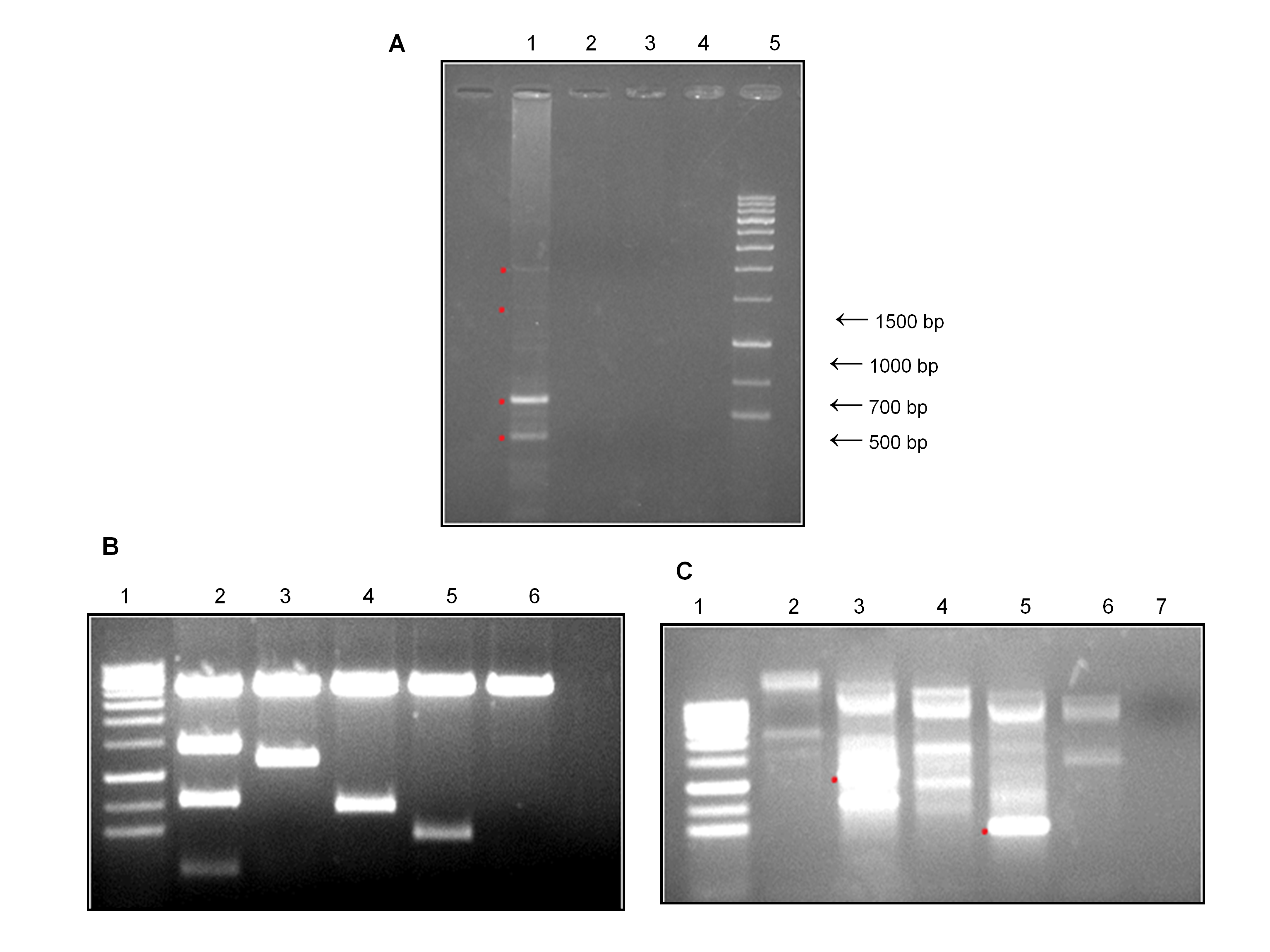

Supplement: Figure S7 — Genome walking in F. oxysporum 11C transformant Tr. 259. Genome walking analysis using APATM Gold Genome Walking Kit (Bio S&T, Canada) to walk the right border (RB) of pSK1019. Lanes represent: 1 - 4; degenerate random tagging (DRT) primers A, B, C and D respectively with target specific primer (GSP) for RB of pSK1019 amplified with 500 ng of fungal genomic DNA from Fusarium oxysporum strain 11C transformant Tr. 259. Arrow indicates PCR fragment sizes (bp) on Hyperladder™ III (Bioline, UK) (Lane 5). Red dots indicate gel excised bands for subsequent cloning (A). Digestion of plasmid DNA from pGEMT ® easy (Promega, UK) cloned PCR products to verify correct fragment insert size. Plasmid DNA (500 ng) was digested in 20 µl reaction and incubated at 37°C for 2 hours. Lanes represent: 1, Hyperladder™ III (Bioline, UK), 2 - 5, bands excised from Figure S7A highlighted by red dots; 6, blue colony (negative control) (B). PCR analysis of cloned PCR products to verify presence of target specific primer (GSP) used in genome walking prior to sequencing. Lanes represent: Lanes represent: 1, Hyperladder™ III (Bioline, UK), 2 - 5, bands excised from Figure S7A highlighted by red dots; 6, blue colony (negative control); 7, no plasmid DNA (negative control) (C). (TIF) [file pone.0077501.s007.tif]

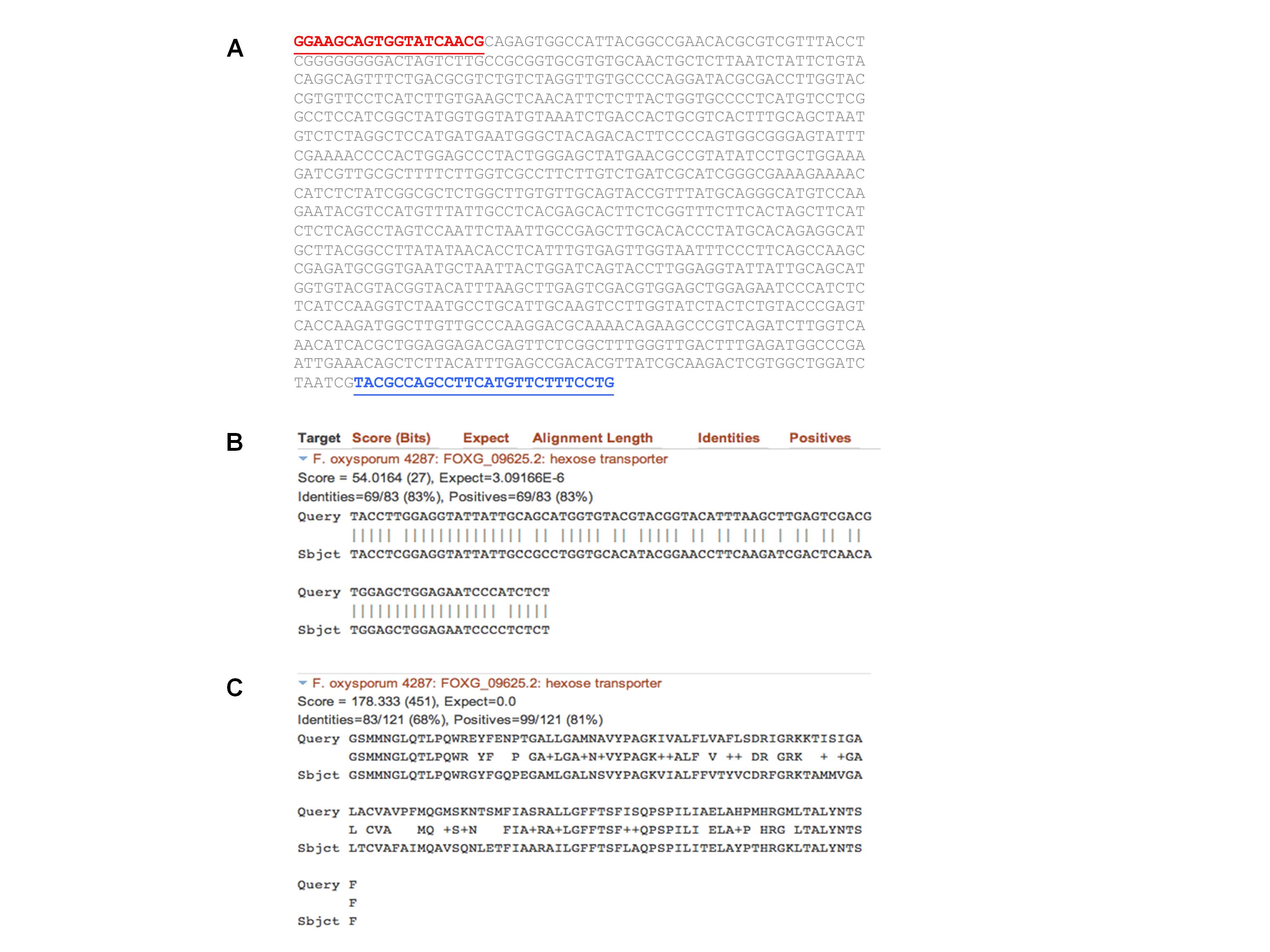

Supplement: Figure S8 — Insilico analysis of identified coding sequence obtained via genome walking in Tr. 259. Sequence analysis of cloned PCR product obtained via genome walking within Fusarium oxysporum strain 11C transformant Tr. 259. Walking was from the right border (RB) of the transformation vector pSK1019 using the APATM Gold Genome Walking Kit (Bio S&T, Canada), a universal kit primer (UAP-2) (red) and the gene-specific (GSP) primer (blue) (3’-5’) (A). Identified coding sequence was analysed against the F. oxysporum f.sp.lycospersici (strain 4287) genome (FCGD; (http://www.broadinstitute.org/annotation/genome/fusarium_group)) using Blastn (B) and Blastx (C). (TIF) [file pone.0077501.s008.tif]
