# Supplementary material for: Generating Phenotypic Diversity in a Fungal Biocatalyst to Investigate Alcohol Stress Tolerance Encountered during Microbial Cellulosic Biofuel Production
Source: PLoS One. 2013 Oct 16;8(10):e77501. doi: 10.1371/journal.pone.0077501 (PMC3797763; doi:10.1371/journal.pone.0077501)
Supplement: Table S1 — Primers used in this study for genome walking and real-time PCR. (DOC) [file pone.0077501.s010.doc]

**Table S1 Primers used in this study for genome walking and real-time PCR**

| **Primer ID** | **Target sequence** | **Forward primer (5'-3')** | **Reverse primer (5'-3')** |
| --- | --- | --- | --- |
| GSP a | right border pSK1019 | TTGAGCTTGGATCAGATTGTCGTTT | - |
| GSP b | right border pSK1019 | CAGCCTGAATGGCGAATGCTAGA | - |
| GSP c | right border pSK1019 | CAGGAAAGAACATGAAGGCTGGCGTA | - |
| SP6^+^ | pGEMT easy vector | ATTTAGGTGACACTATAGAA | - |
| T7^+^ | pGEMT easy vector | TAATACGACTCACTATAGGG | - |
| *Hxt* | hexose transporter (FOXG_09625) | CACATTCGTTGGTCTTGTCG | AATCGCCCATTCTCAATGTC |
| β-tubulin* | housekeeping gene (FOXG_06228) | CAACAACATCCAAACAGCCC | CTCACCAACACGCTTGAAGA |

*[**54**]

^+^pGEMT© Easy vector system (Promega, UK).

Primers were designed using Primer3 software (version 0.4.0; http://frodo.wi.mit.edu/primer3).
