# Supplementary material for: Generating Phenotypic Diversity in a Fungal Biocatalyst to Investigate Alcohol Stress Tolerance Encountered during Microbial Cellulosic Biofuel Production
Source: PLoS One. 2013 Oct 16;8(10):e77501. doi: 10.1371/journal.pone.0077501 (PMC3797763; doi:10.1371/journal.pone.0077501)
Supplement: Methods S1 — Expanded description of methods. (DOCX) [file pone.0077501.s011.docx]

**Methods S1**

ATMT transformation

Fungal conidial inoculum was produced in Mung bean broth [**32**]. *Agrobacterium tumefaciens* AGL-1 harbouring pSK1019 was grown in minimal medium (MM) [**34**] with kanamycin (Apollo Scientific, UK) at a concentration of 50 μg ml^-1^ for 2 days at 28°C 200 rpm. The T-DNA within pSK1019 contains the hygromycin resistant coding *hph* gene under the control of the *Aspergillus nidulans* trpC promoter. The bacterial culture was then inoculated into induction medium (IM) [**34**] containing kanamycin (50 μg ml^-1^) to an OD_600nm_ of 0.15 and grown for 6h at 28°C, 200 rpm. An equal volume of bacterial suspension and conidia (at a concentration of 10^4^ ml^-1^) were mixed and co- cultivated at 28°C, 200 rpm for 30 minutes. A volume of 100 μl was then spread on UV-sterilised cellulose membranes (Maidstone, UK) placed on Petri plates (150x20mm; Sarstedt, Germany) containing co-cultivation medium (IM and 1.5% wv^-1^ agar) [**34**] and plates were incubated for 2 days at 25°C. Following adequate bacterial growth on the plates, cellulose membranes were transferred to modified selection medium (SM) [**71**] that contained (per litre) 50 ml of Macro salt solution (per litre: 60 g KNO_3_, 5.2 g KCl, 5.2g MgSO_4_.7H_2_O, 8.2 g KH_2_PO_4_, 10 g glucose and 2 ml of Micro salt solution (Duchefa® CoCl_2_.6H_2_O, CuSO_4_.H_2_O, FeNaEDTA, H3BO3, KI, MnSO_4_.H_2_O, Na_2_MoO_4_.H_2_O, ZnSO_4_.7H_2_O, KNO_3_; Duchefa, The Netherlands, and 1.5% (wv^-1^) agar technical No.3; Sigma, UK)) supplemented with 60 μg ml^-1^ hygromycinB (Sigma, UK) to select for transformed cells and cefotaxime (Apollo Scientific, UK) and timentin (Apollo Scientific, UK) at a concentration of 400 μg ml^-1^ respectively to kill off residual AGL-1 cells. After 7-9 days, hygromycinB-resistant colonies appearing on the cellulose membrane were transferred to 96-well microtiter plates (Sarstedt, Germany) containing minimal medium [**37**] supplemented with hygromycinB (60 μg ml^-1^) and cefotaxime (200 μg ml^-1^). After a 3-day incubation period at 25°C putative transformants were transferred to a microtiter (96-well) plate assay for primary alcohol screening.

Molecular analysis of transformants

For PCR analysis, fungal genomic DNA was extracted from mycelia using a modified method [**35**]. Frozen (-80^0^C) mycelia-based stock (20% glycerol vv^-1^) (20-50 µl) was inoculated into 2 ml tubes with 1 ml of malt extract (2% wv^-1^ malt) and the tubes were incubated at 25°C, 200 rpm for 5 days and subsequently centrifuged for 15 minutes at 13000 g. Resulting mycelia was transferred to a fresh 1.5 ml tube containing 500μl lysis buffer and the suspension mixed using a sterile cocktail stick before being vortexed for 1 minute. Tubes were left at room temperature for up to 15 minutes, after which 150 μl of potassium acetate solution was added and the tubes were briefly vortexed before being centrifuged for 15 minutes at 13000 g. Approximately 500 μl of the upper phase was then transferred to a fresh 1.5 ml tube containing an equal volume of isopropanol and the tubes incubated overnight at -20°C before being centrifuged for 30 minutes at 13000 g and the supernatant discarded. One ml of 70% ethanol was then added to each tube which was incubated at RT for 1 hour. After centrifuging for 15 minutes at 13000 g, the supernatant was discarded and pellets were left to air dry for 5-10 minutes, before being resuspended in RNAseA water (0.1 mg ml^-1^) and incubated at 37°C for 20-30 minutes. DNA samples were quantified using a Qubit^TM^ Quant-iT assay (Invitrogen, U.S) and stored at -80°C. DNA was quantified using a Qubit^TM^ Quant-iT assay (Invitrogen, U.S) and stored at -80°C. PCR was used to detect the presence/absence of the *hph* transgene in DNA extracts from putative transformants using the primers *hph-F/R*: 5’-gatgttggcgacctcgtatt-3’/5’-gatgtaggagggcgtggata-3’ in 20 μl reactions containing 15 ng of template DNA, 1 X PCR buffer and 1 U Taq DNA polymerase (NEB, UK), 10 mM of each dNTP and 100 nM of the *hph-F* and *hph*-R primer. The initial denaturing cycle was 95°C for 5 min followed by 40 cycles of 95°C for 30 sec, 64.5°C for 45 sec and 72°C for 30 sec and finished with a final extension of 72°C for 5 min. PCR products were visualised on a 1% (wv^-1^) agarose gel which was stained with ethidium bromide (0.5 μl g ml^-1^) and the image captured with a Kodak Imager (Image Station 440CF, Kodak DigitalScienceTM, U.S.).

For Southern blot analysis, genomic DNA was extracted [**36**] and quantified using a NanoDrop® ND-1000 Spectrophotometer (NanoDrop Technologies, USA). Southern blot analysis was used to determine plasmid copy number in transformants. A total of 10 μg of genomic DNA from each transformant and the untransformed *F. oxysporum* wild-type strain 11C and 2 μg of the positive control pSK1019 DNA was digested with *Eco*R1 (NEB, UK) overnight at 37°C. The digested DNA was separated by electrophoresis on a 0.8% (wv^-1^) TAE-agarose gel with 0.5 μg ml^-1^ ethidium bromide. The DNA was blotted to Hybond+ nylon membrane and probed with a 741nt denatured *hph* PCR fragment amplified using *hygB F/R* primers (Ali et al. 2013). The *hygB* DNA probe was labelled using Alkophos direct labelling system and detected using CDP-Star (both labelling and detection were done according to the manufacturer’s recommendations; Amersham, UK). Fluorescence signal was captured using Amersham ECL film following 48 hours exposure.

Primary screening selection of putative transformants for purification

To isolate individuals of interest from the primary screen a threshold was set to divide the population into two groupings: Group I, growth < wild-type strain 11C and Group II, growth 2-fold ≥ wild-type strain 11C. In order to prevent the selection of any individuals with potential growth impediments, putative transformants (*n*=182) that recorded at a minimum ≥ 2-fold increase in growth relative to wild-type strain 11C (Group II) under ethanol stress were purified to monoconidial cultures.

Purification of putative transformants

Putative transformants selected for further study (based on the results of the primary screen) were purified via inoculation onto Spezieller nährstoffarmer agar (SNA) medium [**72**]. Four single spores were isolated per putative transformant to ensure the rescue of a phenotype of interest from a mixed culture. Spores were inoculated into 24-well microtiter plates (Sarstedt, Germany) containing 1.5 ml of minimal medium supplemented with hygromycinB (60 μg ml^-1^). Plates were incubated at 25°C for 5 days; wells were then supplemented with 20% (wv^-1^) glycerol and plates were stored at -80°C.

Tertiary alcohol tolerance screen

The homogeneity of tertiary screen datasets across three replicate experiments was confirmed by one-tailed correlation analysis conducted using Spearman Rank (non-normal data) within SPSS (*r* ≥ 0.582; *P*=0.01) [**73**]. Datasets were therefore pooled for further statistical analysis. Percentage growth of each transformant relative to wild-type control was determined and each treatment analysed individually and depicted using a bar chart representing mean fungal growth and standard error of the mean. Where possible, non-normally distributed data were transformed to fit a normal distribution using Johnson transformation within Minitab (Minitab release © 16, 2011 Minitab Inc.) The significance of treatment effects was analysed within Minitab by either (i) normally distributed data – one-way ANOVA or (ii) non-normally distributed data – the Kruskal-Wallis H-test. To compare the effect of treatments between wild-type 11C and individual transformants, either the paired *t*-test (normally distributed data) or Mann-Whitney test (non-normally distributed data) was conducted within Minitab. Correlation analysis (normal data: Pearson product moment; non-normal data: Spearman Rank) between butanol and ethanol treatments respectively was conducted within SPSS. Principal component analysis (PCA) was used to reduce the dimension of the tertiary dataset comprising of several inter-related variables whilst retaining the variation present within the dataset [**74**]. PCA was conducted on the tertiary dataset (no alcohol, ethanol and butanol treatments on all 29 transformants) using XLSTAT (Addinsoft) v2013.1 software. PCA observation plots were built based on the Scree plot in the factor space according to principal components F1/F2 and F1/F3.

Phenotypic analysis of *F. oxysporum* 11C and Tr. 259

**Effect of increasing alcohol concentration on growth of *F. oxysporum* 11C and Tr. 259**

The tolerance of *F. oxysporum* 11C and transformant Tr. 259 to varying concentrations of both ethanol and butanol was investigated. Microtiter plates (96-well; Sarstedt, Germany) were prepared with 100 µl of the minimal medium [**37**] (per well) supplemented with concentrations of either 0, 2, 4, 6, 8 and 10% (vv^-1^) ethanol (Ethyl Absolute, Sigma, UK) or 0, 0.25, 0.5, 0.75, 1.25, 1.5, 1.75 and 2% (vv^-1^) butanol (*n-*Butanol Butyl alcohol, Sigma, UK). Each microtiter plate contained a single treatment (either ethanol or butanol or no alcohol). To each plate 100 µl of either *F. oxysporum* 11C or transformant Tr. 259 at a concentration of 10^6^ ml^-1^ spores was added. For each treatment, three replicate plates were used with 24 replicates per strain tested per microtiter. Microtiter plates were then sealed with parafilm and incubated at 25°C for 7 days. Fungal growth measured using a spectrophotometer (Spectra Max 340 PC 96-well plate reader, Molecular devices, USA) at OD_600nm_. Each experiment was conducted three times.

**Temporal analysis of alcohol tolerance in *F. oxysporum* 11C and Tr. 259**

The effect of ethanol and butanol on the growth of *F. oxysporum* 11C and transformant Tr. 259 over time was determined. Microtiter (96-well; Sarstedt, Germany) plates were prepared with 100 µl of minimal medium [**37**] and supplemented with either 4% (vv^-1^) ethanol or 0.75% (vv^-1^) butanol as well as a no alcohol control. Each microtiter plate contained a single treatment (alcohol or no alcohol). A microtiter plate was prepared for each time point tested: 24, 48, 72, 96, 120, 144 and 168 hours. To each plate 100 µl of either *F. oxysporum* 11C or transformant Tr. 259 at a concentration of 10^6^ ml^-1^ spores was added. For each treatment, three replicate plates were used with 24 replicates per strain tested per microtiter. Microtiter plates were then sealed with parafilm and incubated at 25°C for 168 hours. Fungal growth measured every 24 hours using a spectrophotometer (Spectra Max 340 PC 96 well plate reader, Molecular devices, USA) at OD_600nm_. Each experiment was conducted twice.

**Effect of alcohol stress on spore germination of *F. oxysporum* 11C and Tr. 259**

The effect of ethanol and butanol on the rate of spore germination in strain 11C and Tr. 259 was investigated. Conical flasks (100 ml) were prepared with 48 ml of minimal medium [**37**] and plugged with non-absorbent cotton wool. To each flask, 2 ml of fungal spores (10^6^ ml^-1^) was added and incubated at 25°C and 150 rpm. To each flask either no alcohol, 4% (vv^-1^) ethanol or 0.75% (vv^-1^) butanol was added after inoculation of fungal spores. At 2, 4, 6, 8, 10 and 12 hours post-fungal spore inoculation, 1 ml of spores were removed and observed microscopically for germination. A total of 100 spores were counted for each treatment (no alcohol, ethanol or butanol) at each time point tested (2, 4, 6, 8, 10, 12 hours). Spores were scored as ‘germinated’ or ‘not germinated’. A spore was considered germinated if the germ tube length was at least as long as spore. Each experiment was conducted three times.

**Biomass production in *F. oxysporum* 11C and Tr. 259 biomass production under alcohol stress**

Erlenmeyer flasks (100 ml) were prepared with minimal medium [**37**] and plugged with cotton wool. To each flask, 2 ml of fungal spores (3x10^6^ ml^-1^) were added and incubated at 25°C shaking at 150 rpm for 12 hours. At 12 hours, flasks were amended with 2 ml water, ethanol or butanol to give a final concentration of 0% (vv^-1^), 4% (vv^-1^) and 0.75% (vv^-1^), respectively. Fungal material was collected at 72, 120 and 168 hours post-treatment. Flasks were removed on ice and transferred to pre-chilled falcon tubes on ice. Fungal material was centrifuged 10 minutes 4000g at 4°C. Fungal material was washed twice with cold sterile distilled water and snap frozen in liquid nitrogen. Samples were then freeze-dried and dry weight measured across three replicates per strain per treatment per time point. Each experiment was conducted twice.

Genome walking

For genome walking, genomic DNA was extracted using a method previously described [**36**] and quantified using a NanoDrop® ND-1000 Spectrophotometer (NanoDrop Technologies, USA). Gene-specific primers were designed for both left and right borders respectively of vector pSK1019 and genome walking was conducted using the APAgene^TM^ Gold Genome Walking Kit (BIO S&T, Canada) according to the manufacturer’s instructions (primers described in Table S1). If PCR was successful, following visualisation of PCR products on 1% (wv^-1^) agarose gel, single amplicons were extracted using QIAquick Gel Extraction Kit (Qiagen, UK) according to the manufacturer’s instructions (Figure S7A). PCR products were then cloned via a pGEMT® Easy Vector system I kit (Promega, UK) according to the manufacturer’s instructions and transformed colonies isolated in 5 ml LB broth supplemented with 50 µg ml^-1^ ampicillin (Apollo Scientific, UK) and incubated overnight at 37°C 220 rpm. Plasmid DNA was extracted using QIAprep Spin Miniprep Kit (Qiagen, UK) according to the manufacturer’s instructions. A total of 500 ng of plasmid DNA was digested for 2 hours at 37°C with *Eco*R1 (NEB, UK) in a 20 µl reaction. Digested plasmid DNA was visualised on a gel to verify the size of cloned inserts by electrophoresis on a 1% (wv^-1^) TAE-agarose gel with 0.5 µg ml^-1^ ethidium bromide (Figure S7B). Positive plasmid containing the correct size insert was verified by PCR analysis for presence of the gene specific primer in a 20 ul reaction with 5X Green GoTaq® Reaction Buffer (Promega, USA), 1.5 mM GoTaq® MgCl_2_ (Promega, USA), 2 mM dNTPs (Sigma, UK) and 100 nM each of either SP6 or T7 (pGEMT easy, Promega, USA) (Table S1) and GSPc and 100 ng of plasmid DNA. PCR program consisted of 94°C 1 min, 94°C 30 sec, 55 °C 30 sec, 72°C 1 min and 72°C 5 min for 35 cycles. PCR products were visualised by electrophoresis on a 1% (wv^-1^) TAE-agarose gel with 0.5 µg ml^-1^ ethidium bromide. PCR positive plasmid DNA was subsequently sequenced for further analysis (Figure S7C). Plasmid DNA was sequenced by GATC (Germany) using SP6 and T7 primers. The program VecScreen (http://www.ncbi.nlm.nih.gov/VecScreen/ VecScreen.html) was used to identify contaminating vector sequences. Nucleotide sequences were queried against (i) Fusarium Comparative Genomics Database (FCGD) (https://www.broadinstitute.org/annotation/ genome/fusarium_group), (ii) National Centre for Biotechnology Information (NCBI) (https://www.ncbi.nlm.nih.gov), (iii) *Saccharomyces* Genome Database (https://www.yeastgenome.org) using BLASTn, BLASTx or BLASTp [**75**] (<https://www.ncbi.nlm.nih.gov>).

RT-PCR of putative hexose transporter (FOXG_09625) under alcohol stress

Erlenmeyer flasks (100 ml) were prepared with minimal medium [**37**] and plugged with cotton wool. To each flask, 2 ml of fungal spores (3x10^6^ ml^-1^) were added and incubated at 25°C shaking at 150 rpm for 12 hours. At 12 hours, flasks were amended with 2ml water, ethanol or butanol to give a final concentration of 0% (vv^-1^), 4% (vv^-1^) and 0.75% (vv^-1^), respectively. Flasks were removed sequentially at three time points: 0.5, 2 12 and 120 hours post-treatment respectively, to ice and fungal material transferred to pre-chilled falcons tubes. Fungal material was centrifuged for 10 minutes at 4000g at 4°C and then washed twice with cold sterile distilled water and snap frozen in liquid nitrogen before storage at -70°C. Each experiment was conducted either three times (no alcohol, ethanol) or twice (butanol) with three replicates per strain per treatment per time point. Samples were flash frozen with liquid nitrogen and homogenised in a mill mixer (MM400, Retsch, Germany) with 2.3 mm steel beads. RNA was isolated [**76**] and DNAse-treated using the Turbo DNA-free Kit (Ambion, USA), according to the manufacturer’s recommendations. RNA quality was visualised by electrophoresis on a 1% (wv^-1^) TAE-agarose gel with 0.5 µg ml^-1^ ethidium bromide and quantified using a NanoDrop® ND-1000 Spectrophotometer (NanoDrop Technologies, USA). Real time RT-PCR analysis was used to analyse the temporal expression pattern of the hexose transporter (*Hxt*) (*Fusarium* database no FOXG_09625) and the housekeeping gene β-tubulin (*Fusarium* database no FOXG_06228) in wild type strain *F. oxysporum* strain 11C and transformant Tr. 259 under normal, ethanol and butanol respectively induced stress conditions. Reverse transcription of total RNA was conducted [**77**], except the primer used was oligo dT_12-18_ (Invitrogen). RT products were diluted to 200 µl and 1 µl was PCR amplified in a 10 µl volume reaction (optimised from manufacturer’s instructions (Takara, Japan)) containing 5 µl Premix *Ex Taq* ™ (Perfect Real Time; Takara, Japan) and 100 nM of each of forward and reverse transcript specific primers (Table S1). PCR reactions were conducted in a Stratagene Mx3000™ real-time PCR machine (Stratagene, USA) and the programme consisted of 1 cycle 95°C 30 sec, 40 cycles 95°C 5 sec, 60°C 20 sec and 1 cycle 95°C 1 min, 55°C 30 sec and 95°C 30 sec according to the manufacturer’s instructions (Takara, Japan). Data was analysed using Stratagene Mx3000™ software (Stratagene, USA). The housekeeping gene β-tubulin (*Fusarium* database no FOXG_06228) was used for normalisation of RT-PCR data. Real-time quantification of the target and housekeeping gene respectively were performed in separate reactions. The threshold cycle (*C*_T_) values obtained from RT-PCR were used to calculate the accumulation of the target gene (relative mRNA accumulation) relative to β-tubulin transcript by the 2^-^*^ΔΔC^*_T_ method, where ΔΔ*C*_T_ = (*C*_T_, target gene – *C*_T_, β-tubulin) [**38**]. Results were based on the average obtained for two replica RT-PCR reactions per sample.

Phylogenetic tree analysis

The protein sequences were aligned using ClustalW and a distance tree of 100 boostrapped datasets was generated. The relationship between these transcripts was examined by the Neighbor-Joining method using Phylogeny.fr software. A second phylogram was constructed as above to compare 13 putative hexose transporters from *F. oxysporum* 4287 and *S. cerevisiae* 20 hexose transporters. Putative sugar transporters showing ≥30% homology to FOXG_09625 in other filamentous fungi were also included in the second phylogram. Amino acid sequences were obtained from FGCD (http://www.broadinstitute.org/annotation/genome/fusarium_group) SGD (http://www.yeastgenome.org) and NCBI (http://www.ncbi.nlm.nih.gov). Pfam (<http://pfam.sanger.ac.uk>) search of all putative sugar transporters used in phylogenetic analysis identified sugar transporter and Major Facilitator Superfamily (MFS) protein domains.

Data analysis for phenotypic and genomic studies in Tr. 259

All datasets were non-normally distributed as determined by the Ryan Joiner test using Minitab (Minitab release 16©, 2011 Minitab Inc.). The homogeneity of the datasets across replicate experiments was confirmed by two–tailed analysis using Spearman Rank for non–normally distributed data conducted with the Statistical Package for the Social Sciences (SPSS 11.0, SPSS Inc.). Datasets showing significant correlation were pooled for further statistical analysis. The significance of treatment effects was analysed within Minitab (Minitab release 16©, 2011 Minitab Inc.) using the non-parametrical Mann-Whitney test. For RT-PCR analysis, data was normally and non-normally distributed as determined by the Ryan Joiner test. Non-normally distributed data was transformed using Johnston transformation to fit a normal distribution within Minitab (Minitab release 16©, 2011 Minitab Inc.). Homogeneity of data sets across replicate experiments was confirmed by two-tailed correlation analysis using Pearson product moment within the Statistical Package for the Social Sciences (SPSS 11.0, SPSS Inc.) (*r* ≥ 0.844; *P*=0.001). Datasets from replicate experiments were pooled for further analysis. Significance of treatment effects was analysed within Minitab (Minitab release 16©, 2011 Minitab Inc.) using One-way ANOVA with Post–HOC Tukey’s HSD test. Statistical differences of two means were calculated using the paired *t*-test function within Minitab (Minitab release 16©, 2011 Minitab Inc.). Correlation analysis (normal data: Pearson product moment; non-normal data: Spearman Rank) between mean fungal growth/ ethanol concentrations, fungal growth/ butanol concentrations and mean spore germination/time were conducted within SPSS.
