# Supplementary material for: Generating Phenotypic Diversity in a Fungal Biocatalyst to Investigate Alcohol Stress Tolerance Encountered during Microbial Cellulosic Biofuel Production
Source: PLoS One. 2013 Oct 16;8(10):e77501. doi: 10.1371/journal.pone.0077501 (PMC3797763; doi:10.1371/journal.pone.0077501)
Supplement: References S1 — (DOCX) [file pone.0077501.s012.docx]

Supplementary References

1. Tzfira T, Frankman L, Vaidya M, Citovsky V (2003) Site-specific integration of *Agrobacterium* T-DNA via double-stranded intermediates. Plant Physiology. 133: 1011-1023.
2. Khang CH, Park S-Y, Lee Y-H, Kang S (2005) A dual selection based, targeted gene replacement tool for *Magnaporthe grisea* and *Fusarium* *oxysporum*. Fungal Genetics and Biology. 42(6): 483-492.
3. Leslie JF, Summerell BA (2007) The *Fusarium* Laboratory Manual.
4. Snedecor G, Cochran W (1980) Statistical methods, 6th edn. Iowa State University Press, Ames
5. [Bhatta H](http://www.ncbi.nlm.nih.gov/pubmed?term=Bhatta%20H%5BAuthor%5D&cauthor=true&cauthor_uid=16437201), [Goldys EM](http://www.ncbi.nlm.nih.gov/pubmed?term=Goldys%20EM%5BAuthor%5D&cauthor=true&cauthor_uid=16437201), [Learmonth RP](http://www.ncbi.nlm.nih.gov/pubmed?term=Learmonth%20RP%5BAuthor%5D&cauthor=true&cauthor_uid=16437201) (2006) Use of fluorescence spectroscopy to differentiate yeast and bacterial cells. Appl Microbiol Biotechnol. 71(1): 121-6.
6. Altschul SF, Madden TL, Schäffer AA, Zhang J, Zhang Z, et al. (1997) Gapped BLAST and PSI-BLAST: a new generation of protein database search programs. *Nucleic Acids Res*. 25(17): 3389– 3402
7. Chang S, Puryear J, Cairney J (1993) A simple and efficient method for isolating RNA from pine trees. *Plant Molecular Biology Reporter* 11(2): 113-116.
8. Ansari KI, Walter S, Brennan JM, Lemmens M, Kessans S, McGahern A et al. (2007) Retrotransposon and gene activation in wheat in response to mycotoxigenic and non-mycotoxigenic-associated *Fusarium* stress. *TAG Theoretical and Applied Genetics* 114(5): 927-937.
